# Supplementary material for: Analysis of Efficacy-To-Safety Ratio of Angiogenesis-Inhibitors Based Therapies in Ovarian Cancer: A Systematic Review and Meta-Analysis
Source: Diagnostics (Basel). 2023 Mar 9;13(6):1040. doi: 10.3390/diagnostics13061040 (PMC10046967; doi:10.3390/diagnostics13061040)
Supplement: Supplementary file 1 [file diagnostics-13-01040-s001.zip › diagnostics-2218672-supplementary.pdf]

Supplementary Material 1 Figures S1-S31 v.3.0

Figure S1: Hypertension

	Experimental		Control			Risk Ratio
**Study or Subgroup**	**Events**	**Total**	**Events**	**Total**	**Weight**	**M-H, Random, 95% CI**
Aghajanian (OCEANS)	104	247	20	233	6.9%	4.91 [3.15, 7.65]
Coleman (GOG0213)	135	330	10	327	6.8%	13.38 [7.17, 24.96]
du Bois	129	902	25	450	6.9%	2.57 [1.70, 3.89]
Gore	19	26	11	24	6.8%	1.59 [0.97, 2.61]
Gotlieb	5	30	0	25	4.4%	9.23 [0.53, 159.14]
Herzog	45	123	7	123	6.7%	6.43 [3.02, 13.69]
Karlan	7	105	3	55	6.2%	1.22 [0.33, 4.54]
Kim (East Asian Study)	117	179	34	174	6.9%	3.35 [2.43, 4.61]
Ledermann (ICON6)	38	95	42	115	6.9%	1.10 [0.78, 1.55]
Liu	34	44	0	46	4.5%	72.07 [4.55, 1140.62]
Marth (TRINOVA-2)	12	113	9	108	6.6%	1.27 [0.56, 2.90]
Oza (ICON 7)	46	745	2	753	6.1%	23.25 [5.66, 95.42]
Pignata (MITO 11)	16	37	0	36	4.4%	32.13 [2.00, 516.29]
Pignata (MITO16B)	197	201	186	200	7.0%	1.05 [1.01, 1.10]
Pujade-Lauraine (AURELIA)	13	179	2	181	6.0%	6.57 [1.50, 28.71]
Vergote (AGO-OVAR16)	275	477	91	461	7.0%	2.92 [2.39, 3.57]
**Total (95% CI)**		**3833**		**3311**	**100.0%**	**4.26 [1.61, 11.26]**
Total events	1192		442			

Heterogeneity: Tau² = 3.53; Chi² = 1690.13, df = 15 (P < 0.00001); I² = 99%

Test for overall effect: Z = 2.92 (P = 0.003)

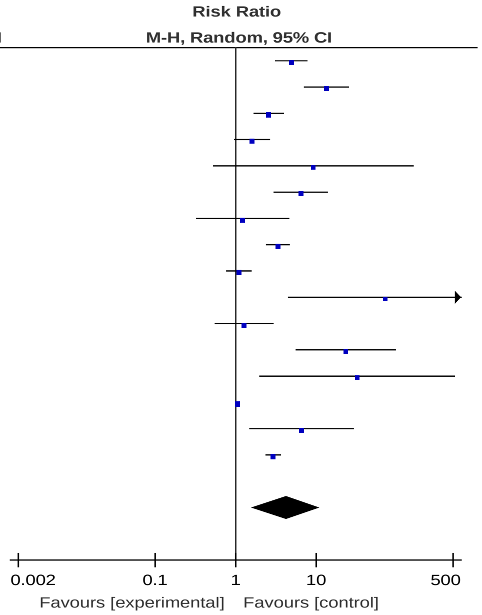

Figure S2: Hemorrhagic events

Study or Subgroup	Experimental Events	Experimental Total	Control Events	Control Total	Weight	Risk Ratio M-H, Random, 95% CI
Chekerov (TRIAS)	7	83	6	89	13.1%	1.25 [0.44, 3.57]
Coleman (GOG0213)	140	330	27	327	18.3%	5.14 [3.50, 7.53]
Gore	7	26	2	24	10.0%	3.23 [0.74, 14.06]
Gotlieb	3	30	2	25	8.5%	1.25 [0.23, 6.90]
Karlan	28	105	13	55	17.0%	1.13 [0.64, 2.00]
Ledermann (ICON6)	15	95	15	115	16.3%	1.21 [0.62, 2.35]
Oza (ICON 7)	9	745	2	753	9.6%	4.55 [0.99, 20.98]
Pujade-Lauraine (AURELIA)	2	179	2	181	7.3%	1.01 [0.14, 7.10]
**Total (95% CI)**		**1593**		**1569**	**100.0%**	**1.94 [1.00, 3.76]**
Total events	211		69			

Heterogeneity: Tau² = 0.59; Chi² = 30.27, df = 7 (P < 0.0001); I² = 77%

Test for overall effect: Z = 1.95 (P = 0.05)

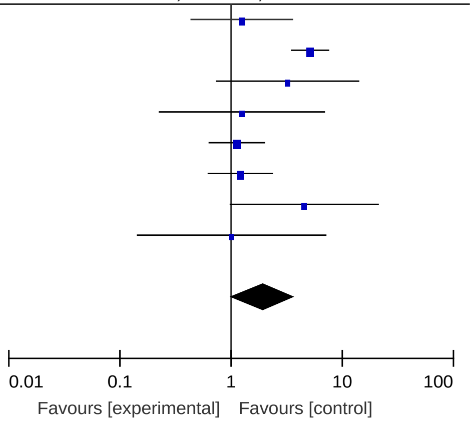

Figure S3: Thrombosis or embolism

Study or Subgroup	Experimental Events	Experimental Total	Control Events	Control Total	Weight	Risk Ratio M-H, Fixed, 95% CI
Chekerov (TRIAS)	4	83	6	89	6.9%	0.71 [0.21, 2.44]
du Bois	70	902	29	450	46.2%	1.20 [0.79, 1.83]
Gore	1	26	0	24	0.6%	2.78 [0.12, 65.08]
Ledermann (ICON6)	3	95	1	115	1.1%	3.63 [0.38, 34.35]
Oza (ICON 7)	51	745	23	753	27.3%	2.24 [1.38, 3.63]
Pignata (MITO 11)	1	37	2	36	2.4%	0.49 [0.05, 5.13]
Pignata (MITO16B)	6	201	5	200	6.0%	1.19 [0.37, 3.85]
Pujade-Lauraine (AURELIA)	9	179	8	181	9.5%	1.14 [0.45, 2.88]
**Total (95% CI)**		**2268**		**1848**	**100.0%**	**1.47 [1.12, 1.92]**
Total events	145		74			

Heterogeneity: Chi² = 7.17, df = 7 (P = 0.41); I² = 2%

Test for overall effect: Z = 2.75 (P = 0.006)

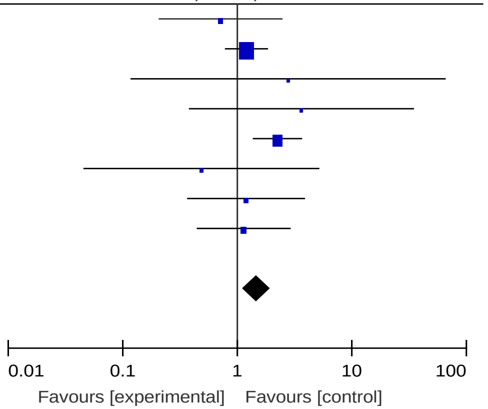

Figure S4: Arterial thromboembolism

	Experimental		Control			Risk Ratio
**Study or Subgroup**	**Events**	**Total**	**Events**	**Total**	**Weight**	**M-H, Fixed, 95% CI**
Aghajanian (OCEANS)	6	247	1	233	3.7%	5.66 [0.69, 46.66]
Coleman (GOG0213)	22	330	6	327	21.9%	3.63 [1.49, 8.84]
du Bois	9	902	2	450	9.7%	2.25 [0.49, 10.35]
Karlan	2	105	0	55	2.4%	2.64 [0.13, 54.08]
Oza (ICON 7)	20	745	10	753	36.2%	2.02 [0.95, 4.29]
Pujade-Lauraine (AURELIA)	4	179	0	181	1.8%	9.10 [0.49, 167.79]
Tewari	8	1215	5	601	24.3%	0.79 [0.26, 2.41]
**Total (95% CI)**		**3723**		**2600**	**100.0%**	**2.38 [1.52, 3.70]**
Total events	71		24			
Heterogeneity: Chi² = 6.28, df = 6 (P = 0.39); I² = 4%						
Test for overall effect: Z = 3.82 (P = 0.0001)						

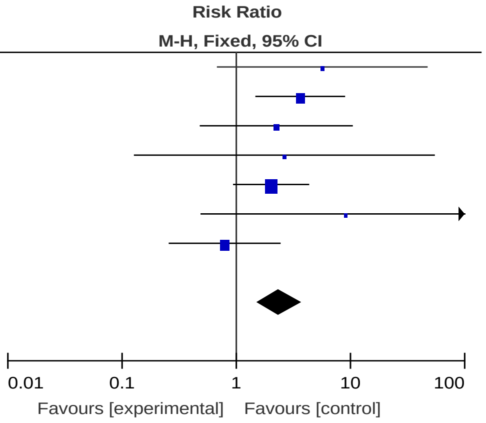

Figure S5: Venous thromboembolism

	Experimental		Control			Risk Ratio
**Study or Subgroup**	**Events**	**Total**	**Events**	**Total**	**Weight**	**M-H, Random, 95% CI**
Aghajanian (OCEANS)	11	247	6	233	11.7%	1.73 [0.65, 4.60]
Coleman (GOG0213)	0	330	0	327		Not estimable
du Bois	33	902	22	450	21.5%	0.75 [0.44, 1.27]
Gotlieb	2	30	0	25	1.9%	4.19 [0.21, 83.50]
Karlan	7	105	6	55	10.8%	0.61 [0.22, 1.73]
Oza (ICON 7)	32	745	13	753	18.6%	2.49 [1.32, 4.70]
Pujade-Lauraine (AURELIA)	5	179	8	181	10.1%	0.63 [0.21, 1.89]
Tewari	78	1215	35	601	25.5%	1.10 [0.75, 1.62]
**Total (95% CI)**		**3753**		**2625**	**100.0%**	**1.13 [0.74, 1.72]**
Total events	168		90			
Heterogeneity: Tau² = 0.14; Chi² = 12.19, df = 6 (P = 0.06); I² = 51%						
Test for overall effect: Z = 0.58 (P = 0.56)						

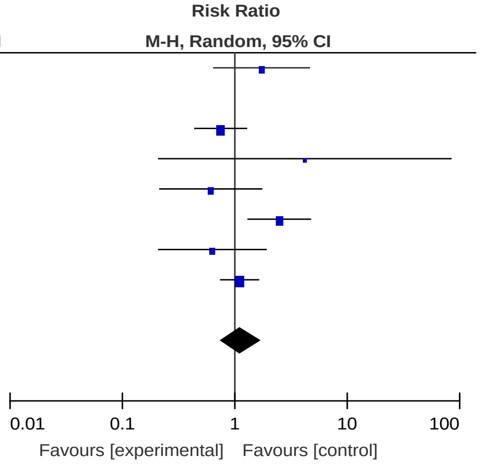

Figure S6: Proteinuria

Study or Subgroup	Experimental Events	Experimental Total	Control Events	Control Total	Weight	Risk Ratio M-H, Random, 95% CI
Aghajanian (OCEANS)	27	247	2	233	8.8%	12.73 [3.06, 52.96]
Coleman (GOG0213)	56	330	3	327	9.6%	18.50 [5.85, 58.50]
Gotlieb	18	30	16	25	11.4%	0.94 [0.62, 1.42]
Karlan	7	105	2	55	8.4%	1.83 [0.39, 8.53]
Kim (East Asian Study)	26	179	4	174	10.0%	6.32 [2.25, 17.73]
Ledermann (ICON6)	16	95	11	115	10.8%	1.76 [0.86, 3.61]
Liu	5	44	0	46	5.0%	11.49 [0.65, 201.83]
Pignata (MITO16B)	82	201	7	200	10.7%	11.66 [5.53, 24.59]
Pujade-Lauraine (AURELIA)	3	179	0	181	4.8%	7.08 [0.37, 136.04]
Tewari	16	1215	4	601	9.8%	1.98 [0.66, 5.89]
Vergote (AGO-OVAR16)	40	477	8	461	10.7%	4.83 [2.29, 10.21]
**Total (95% CI)**		**3102**		**2418**	**100.0%**	**4.52 [1.93, 10.55]**
Total events	296		57			

Heterogeneity: Tau² = 1.60; Chi² = 83.41, df = 10 (P < 0.00001); I² = 88%
Test for overall effect: Z = 3.48 (P = 0.0005)

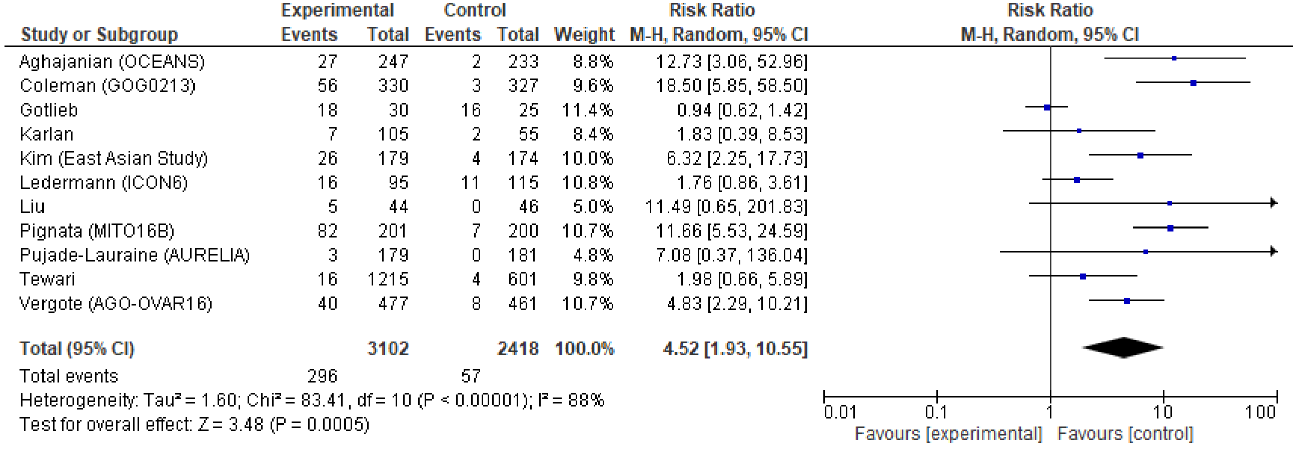

Figure S7: Reversible posterior leukoencephalopathy syndrome

Study or Subgroup	Experimental Events	Experimental Total	Control Events	Control Total	Weight	Risk Ratio M-H, Fixed, 95% CI
Coleman (GOG0213)	0	330	0	327		Not estimable
Pujade-Lauraine (AURELIA)	1	179	0	181	27.1%	3.03 [0.12, 73.97]
Tewari	1	1215	1	601	72.9%	0.49 [0.03, 7.89]
**Total (95% CI)**		**1724**		**1109**	**100.0%**	**1.18 [0.18, 7.94]**
Total events	2		1			

Heterogeneity: Chi² = 0.71, df = 1 (P = 0.40); I² = 0%
Test for overall effect: Z = 0.17 (P = 0.86)

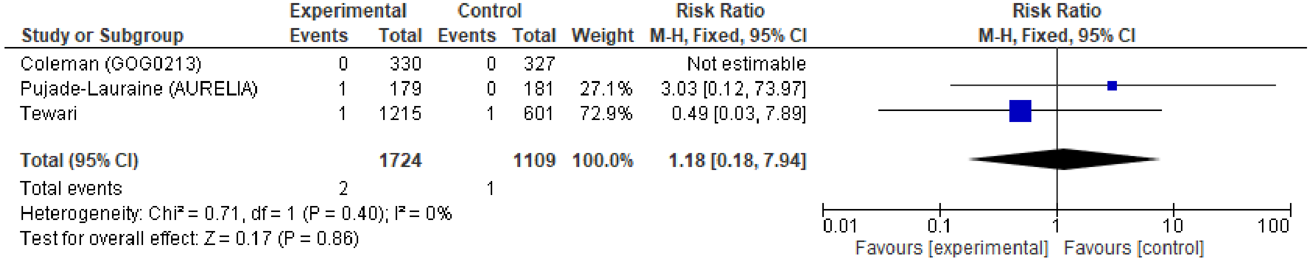

Figure S8: Gastrointestinal perforations

Study or Subgroup	Experimental Events	Experimental Total	Control Events	Control Total	Weight	Risk Ratio M-H, Fixed, 95% CI
Aghajanian (OCEANS)	2	247	1	233	11.3%	1.89 [0.17, 20.67]
Coleman (GOG0213)	6	330	1	327	11.1%	5.95 [0.72, 49.11]
Gore	1	26	0	24	5.7%	2.78 [0.12, 65.08]
Gotlieb	3	30	1	25	12.0%	2.50 [0.28, 22.56]
Karlan	0	105	1	55	21.6%	0.18 [0.01, 4.25]
Oza (ICON 7)	10	745	3	753	32.8%	3.37 [0.93, 12.19]
Pujade-Lauraine (AURELIA)	3	179	0	181	5.5%	7.08 [0.37, 136.04]
**Total (95% CI)**		**1662**		**1598**	**100.0%**	**2.86 [1.36, 6.04]**
Total events	25		7			

Heterogeneity: Chi² = 3.96, df = 6 (P = 0.68); I² = 0%
Test for overall effect: Z = 2.76 (P = 0.006)

Risk Ratio
M-H, Fixed, 95% CI

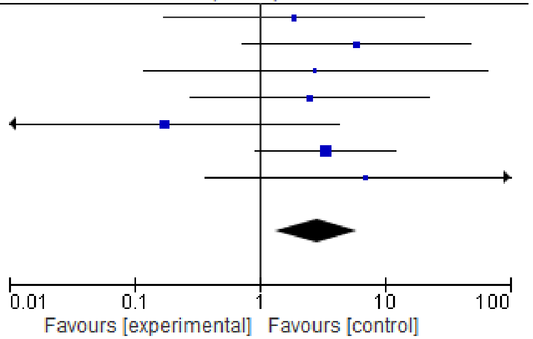

Figure S9: Infection

Study or Subgroup	Experimental Events	Experimental Total	Control Events	Control Total	Weight	Risk Ratio M-H, Fixed, 95% CI
Chekerov (TRIAS)	31	83	31	89	20.8%	1.07 [0.72, 1.60]
Coleman (GOG0213)	126	330	89	327	62.3%	1.40 [1.12, 1.76]
Gore	6	26	4	24	2.9%	1.38 [0.44, 4.32]
Pignata (MITO 11)	5	37	3	36	2.1%	1.62 [0.42, 6.29]
Richardson	15	50	17	50	11.8%	0.88 [0.50, 1.57]
**Total (95% CI)**		**526**		**526**	**100.0%**	**1.28 [1.07, 1.53]**
Total events	183		144			

Heterogeneity: Chi² = 3.15, df = 4 (P = 0.53); I² = 0%
Test for overall effect: Z = 2.65 (P = 0.008)

Risk Ratio
M-H, Fixed, 95% CI

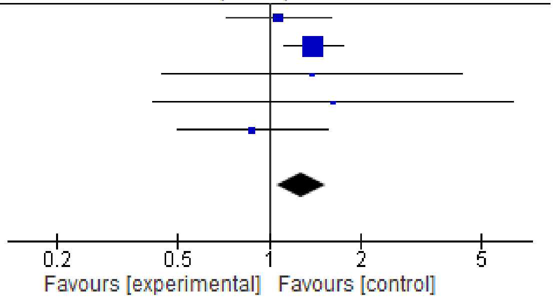

Figure S10: Pyrexia

Study or Subgroup	Experimental Events	Experimental Total	Control Events	Control Total	Weight	Risk Ratio M-H, Fixed, 95% CI
Gore	3	26	3	24	2.8%	0.92 [0.21, 4.14]
Herzog	13	123	10	123	9.1%	1.30 [0.59, 2.85]
Marth (TRINOVA-2)	20	113	18	108	16.7%	1.06 [0.59, 1.90]
Pignata (MITO16B)	23	201	16	200	14.5%	1.43 [0.78, 2.63]
Vergote (TRINOVA-3)	72	675	47	336	56.9%	0.76 [0.54, 1.08]
**Total (95% CI)**		**1138**		**791**	**100.0%**	**0.96 [0.75, 1.23]**
Total events	131		94			

Heterogeneity: Chi² = 4.08, df = 4 (P = 0.40); I² = 2%
Test for overall effect: Z = 0.30 (P = 0.76)

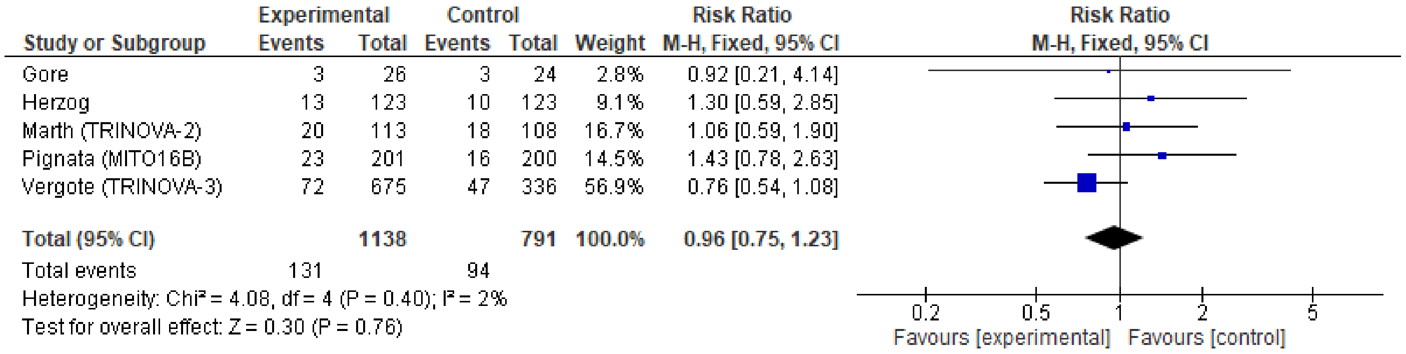

Figure S11: Wound related issues

Study or Subgroup	Experimental Events	Experimental Total	Control Events	Control Total	Weight	Risk Ratio M-H, Fixed, 95% CI
Aghajanian (OCEANS)	2	247	0	233	2.0%	4.72 [0.23, 97.75]
Coleman (GOG0213)	10	330	2	327	7.7%	4.95 [1.09, 22.44]
Karlan	2	105	0	55	2.5%	2.64 [0.13, 54.08]
Pujade-Lauraine (AURELIA)	0	179	0	181		Not estimable
Tewari	40	1215	17	601	87.7%	1.16 [0.67, 2.04]
**Total (95% CI)**		**2076**		**1397**	**100.0%**	**1.57 [0.96, 2.56]**
Total events	54		19			

Heterogeneity: Chi² = 3.94, df = 3 (P = 0.27); I² = 24%
Test for overall effect: Z = 1.78 (P = 0.07)

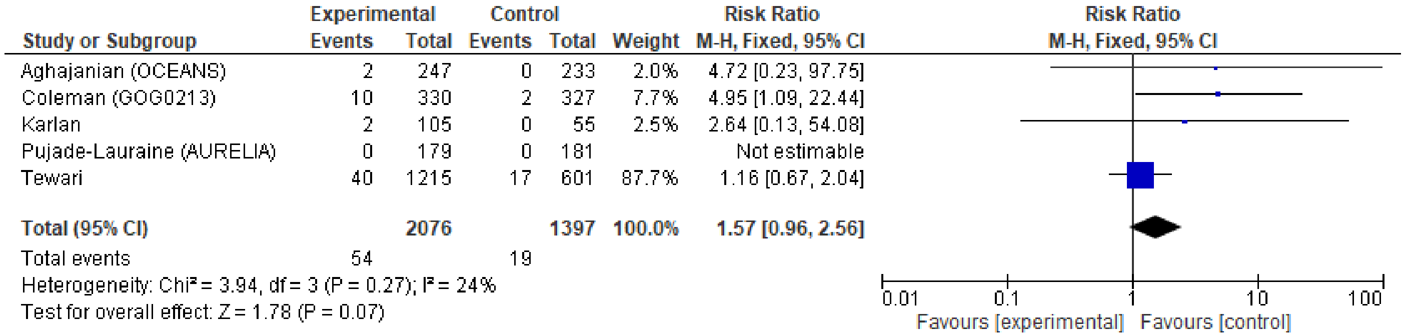

Figure S12: Ascites

Study or Subgroup	Experimental Events	Experimental Total	Control Events	Control Total	Weight	Risk Ratio M-H, Fixed, 95% CI
Marth (TRINOVA-2)	33	113	10	108	9.7%	3.15 [1.64, 6.08]
Monk (TRINOVA-1)	101	461	57	452	54.7%	1.74 [1.29, 2.34]
Vergote (TRINOVA-3)	127	675	28	336	35.5%	2.26 [1.53, 3.33]
**Total (95% CI)**		**1249**		**896**	**100.0%**	**2.06 [1.65, 2.57]**
Total events	261		95			

Heterogeneity: Chi² = 3.09, df = 2 (P = 0.21); I² = 35%
Test for overall effect: Z = 6.37 (P < 0.00001)

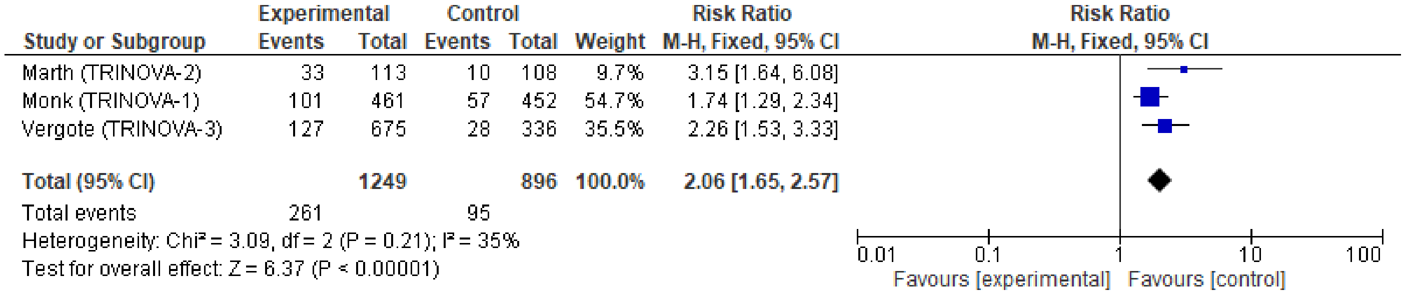

Figure S13: Neutropenia

Study or Subgroup	Experimental Events	Experimental Total	Control Events	Control Total	Weight	Risk Ratio M-H, Random, 95% CI
Aghajanian (OCEANS)	52	247	51	233	5.6%	0.96 [0.68, 1.35]
Chekerov (TRIAS)	52	83	56	89	6.7%	1.00 [0.79, 1.25]
Coleman (GOG0213)	40	330	26	327	4.5%	1.52 [0.95, 2.44]
du Bois	498	902	226	450	7.6%	1.10 [0.99, 1.23]
Gore	11	26	12	24	3.5%	0.85 [0.46, 1.54]
Herzog	3	123	2	123	0.7%	1.50 [0.26, 8.82]
Kim (East Asian Study)	86	179	26	174	5.2%	3.22 [2.19, 4.73]
Ledermann (ICON6)	28	95	55	115	5.4%	0.62 [0.43, 0.89]
Liu	4	44	5	46	1.2%	0.84 [0.24, 2.91]
Marth (TRINOVA-2)	15	113	22	108	3.5%	0.65 [0.36, 1.19]
Monk (TRINOVA-1)	104	461	129	452	6.7%	0.79 [0.63, 0.99]
Oza (ICON 7)	123	745	114	753	6.6%	1.09 [0.86, 1.38]
Pignata (MITO 11)	28	37	11	36	4.0%	2.48 [1.46, 4.19]
Pignata (MITO16B)	147	201	130	200	7.5%	1.13 [0.99, 1.28]
Pujade-Lauraine (AURELIA)	29	179	31	181	4.5%	0.95 [0.60, 1.50]
Richardson	41	50	23	50	5.7%	1.78 [1.29, 2.47]
Tewari	770	1215	347	601	7.7%	1.10 [1.01, 1.19]
Vergote (AGO-OVAR16)	151	477	36	461	5.6%	4.05 [2.88, 5.70]
Vergote (TRINOVA-3)	526	675	282	336	7.8%	0.93 [0.87, 0.99]
**Total (95% CI)**		**6182**		**4759**	**100.0%**	**1.20 [1.03, 1.40]**
Total events	2708		1584			

Heterogeneity: $Tau^2 = 0.07$; $Chi^2 = 165.51$, $df = 18$ ($P < 0.00001$); $I^2 = 89\%$
Test for overall effect: $Z = 2.40$ ($P = 0.02$)

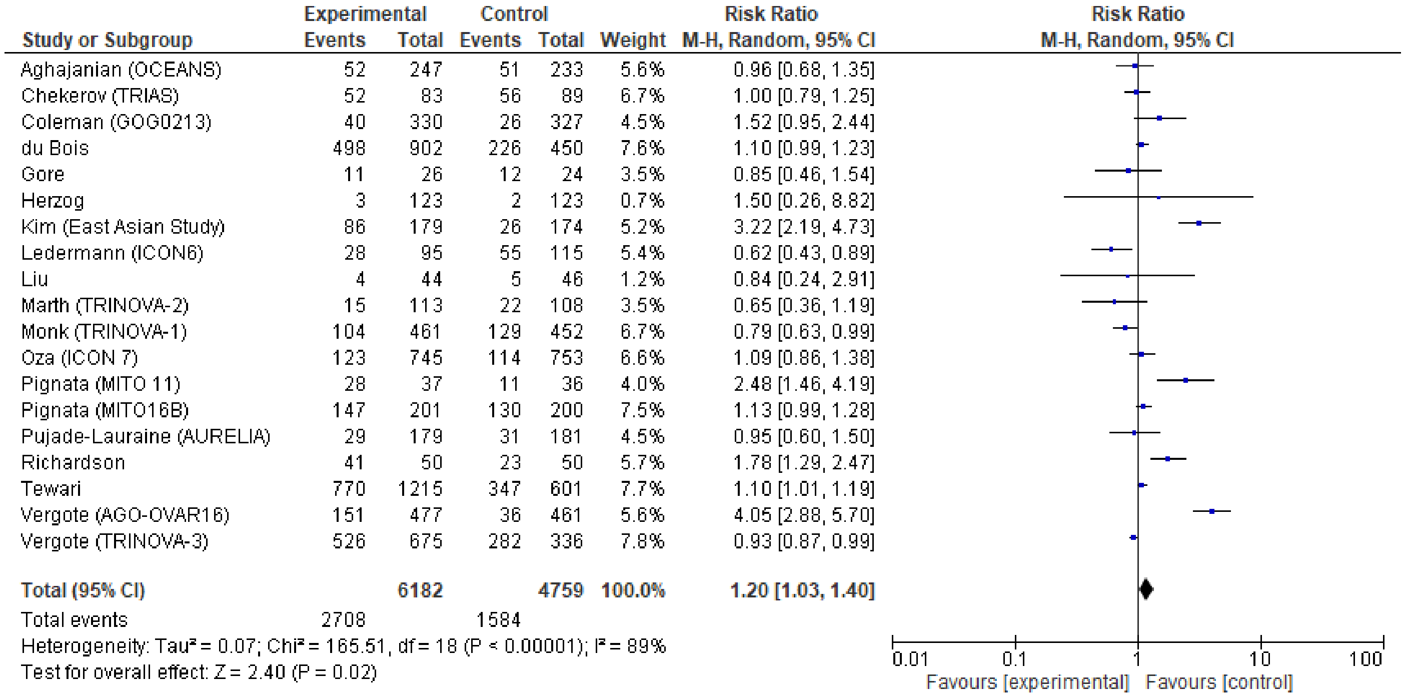

Figure S14: Anemia

Study or Subgroup	Experimental Events	Experimental Total	Control Events	Control Total	Weight	Risk Ratio M-H, Random, 95% CI
Chekerov (TRIAS)	66	83	79	89	17.4%	0.90 [0.79, 1.02]
Gore	10	26	19	24	5.3%	0.49 [0.29, 0.82]
Ledermann (BIBF 1120)	0	43	0	40		Not estimable
Liu	7	44	8	46	2.1%	0.91 [0.36, 2.31]
Marth (TRINOVA-2)	11	113	15	108	3.1%	0.70 [0.34, 1.46]
Monk (TRINOVA-1)	50	461	94	452	10.1%	0.52 [0.38, 0.72]
Pignata (MITO 11)	25	37	22	36	9.3%	1.11 [0.78, 1.56]
Pignata (MITO16B)	134	201	137	200	17.3%	0.97 [0.85, 1.11]
Pujade-Lauraine (AURELIA)	3	179	4	181	0.9%	0.76 [0.17, 3.34]
Richardson	46	50	46	50	18.1%	1.00 [0.89, 1.12]
Vergote (TRINOVA-3)	261	675	146	336	16.5%	0.89 [0.76, 1.04]
**Total (95% CI)**		**1912**		**1562**	**100.0%**	**0.86 [0.75, 0.99]**
Total events	613		570			

Heterogeneity: $Tau^2 = 0.03$; $Chi^2 = 28.27$, df = 9 (P = 0.0009); $I^2 = 68\%$
Test for overall effect: Z = 2.13 (P = 0.03)

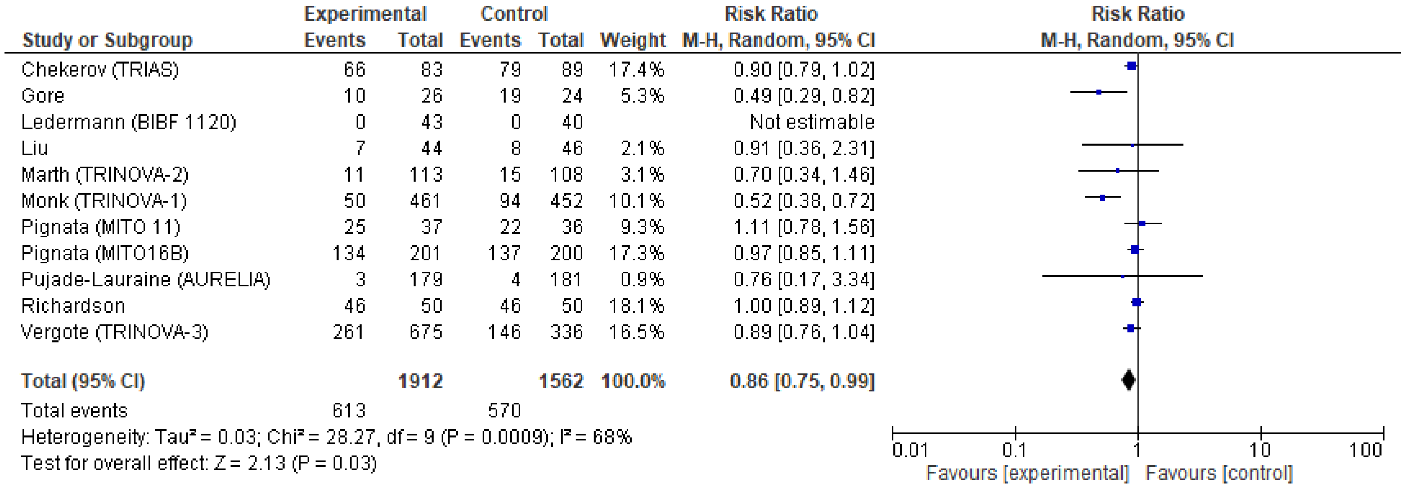

Figure S15: Leucopenia

Study or Subgroup	Experimental Events	Experimental Total	Control Events	Control Total	Weight	Risk Ratio M-H, Random, 95% CI
Chekerov (TRIAS)	66	83	79	89	17.4%	0.90 [0.79, 1.02]
Gore	10	26	19	24	5.3%	0.49 [0.29, 0.82]
Ledermann (BIBF 1120)	0	43	0	40		Not estimable
Liu	7	44	8	46	2.1%	0.91 [0.36, 2.31]
Marth (TRINOVA-2)	11	113	15	108	3.1%	0.70 [0.34, 1.46]
Monk (TRINOVA-1)	50	461	94	452	10.1%	0.52 [0.38, 0.72]
Pignata (MITO 11)	25	37	22	36	9.3%	1.11 [0.78, 1.56]
Pignata (MITO16B)	134	201	137	200	17.3%	0.97 [0.85, 1.11]
Pujade-Lauraine (AURELIA)	3	179	4	181	0.9%	0.76 [0.17, 3.34]
Richardson	46	50	46	50	18.1%	1.00 [0.89, 1.12]
Vergote (TRINOVA-3)	261	675	146	336	16.5%	0.89 [0.76, 1.04]
**Total (95% CI)**		**1912**		**1562**	**100.0%**	**0.86 [0.75, 0.99]**
Total events	613		570			

Heterogeneity: $Tau^2 = 0.03$; $Chi^2 = 28.27$, df = 9 (P = 0.0009); $I^2 = 68\%$
Test for overall effect: Z = 2.13 (P = 0.03)

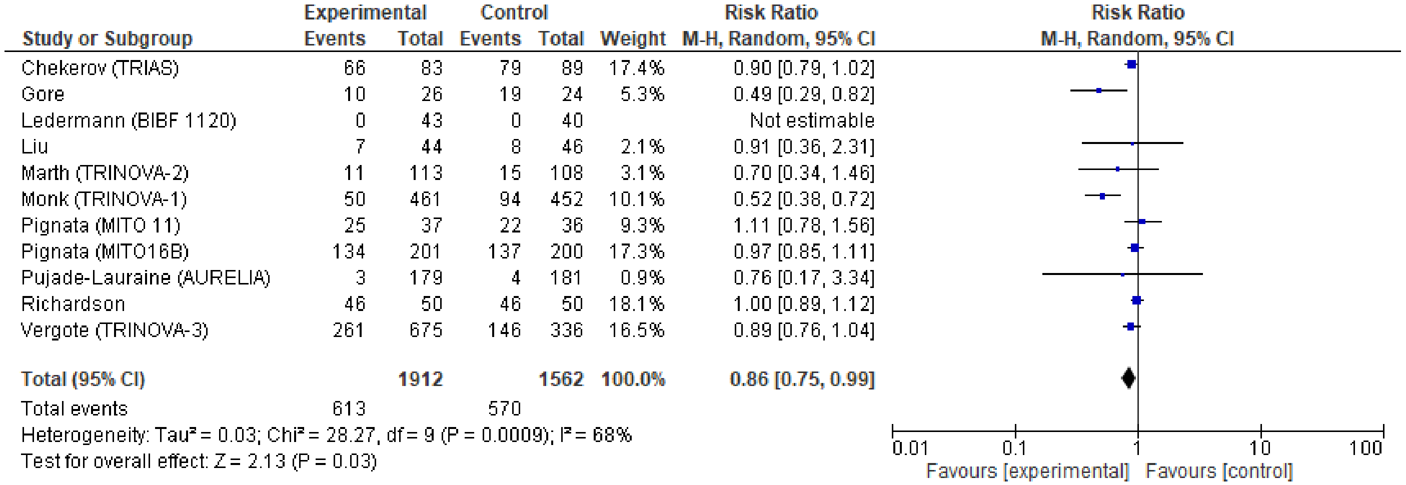

Figure S16: Thrombocytopenia

Study or Subgroup	Experimental Events	Experimental Total	Control Events	Control Total	Weight	Risk Ratio M-H, Random, 95% CI
Aghajanian (OCEANS)	143	247	119	233	11.0%	1.13 [0.96, 1.34]
Chekerov (TRIAS)	62	83	47	89	10.5%	1.41 [1.12, 1.79]
du Bois	363	902	103	450	10.8%	1.76 [1.46, 2.12]
Gore	12	26	11	24	6.6%	1.01 [0.55, 1.84]
Kim (East Asian Study)	39	179	10	174	6.1%	3.79 [1.95, 7.35]
Ledermann (ICON6)	39	95	20	115	8.0%	2.36 [1.48, 3.76]
Liu	8	44	3	46	2.7%	2.79 [0.79, 9.84]
Oza (ICON 7)	26	745	15	753	6.4%	1.75 [0.94, 3.28]
Pignata (MITO 11)	5	37	3	36	2.4%	1.62 [0.42, 6.29]
Pignata (MITO16B)	126	201	104	200	11.0%	1.21 [1.02, 1.43]
Pujade-Lauraine (AURELIA)	3	179	4	181	2.1%	0.76 [0.17, 3.34]
Richardson	24	50	10	50	6.4%	2.40 [1.28, 4.48]
Vergote (AGO-OVAR16)	80	477	9	461	6.0%	8.59 [4.36, 16.91]
Vergote (TRINOVA-3)	184	675	52	336	10.0%	1.76 [1.33, 2.33]
**Total (95% CI)**		**3940**		**3148**	**100.0%**	**1.81 [1.43, 2.29]**
Total events	1114		510			

Heterogeneity: Tau² = 0.13; Chi² = 72.44, df = 13 (P < 0.00001); I² = 82%
Test for overall effect: Z = 4.91 (P < 0.00001)

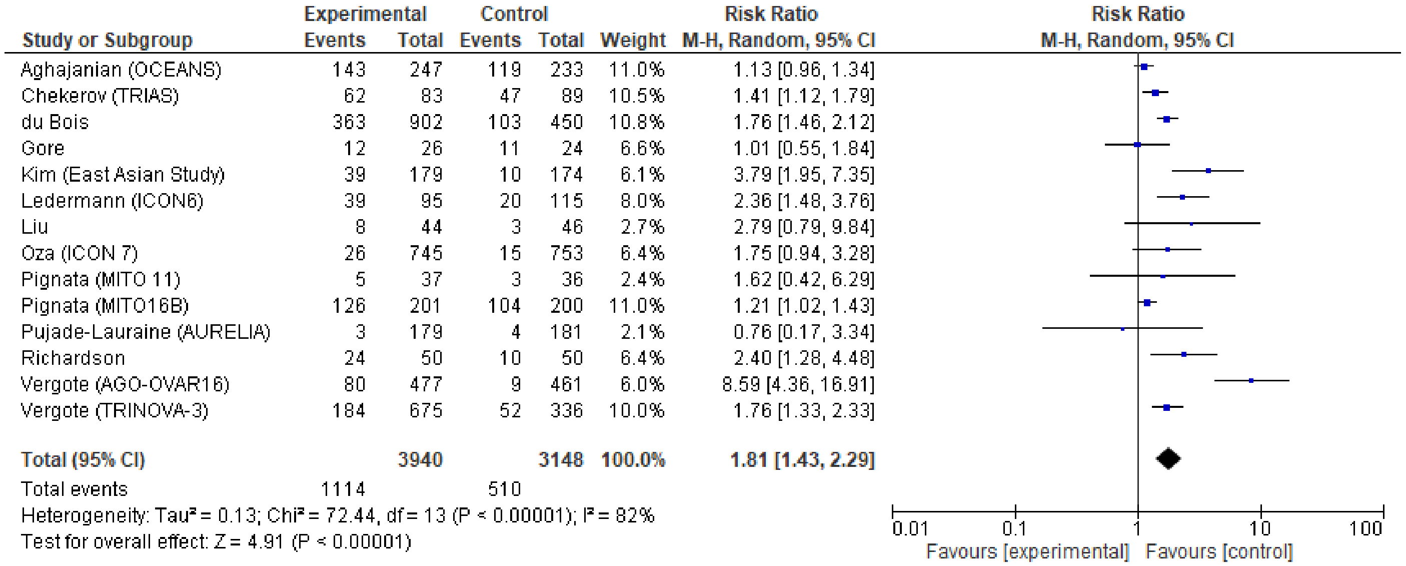

Figure S17: Nausea

Study or Subgroup	Experimental Events	Experimental Total	Control Events	Control Total	Weight	Risk Ratio M-H, Random, 95% CI
Aghajanian (OCEANS)	179	247	153	233	10.8%	1.10 [0.98, 1.24]
Chekerov (TRIAS)	51	83	50	89	8.8%	1.09 [0.85, 1.40]
du Bois	586	902	236	450	11.0%	1.24 [1.12, 1.37]
Gotlieb	9	30	7	25	2.6%	1.07 [0.47, 2.46]
Herzog	33	123	26	123	5.8%	1.27 [0.81, 1.99]
Karlan	58	105	17	55	6.1%	1.79 [1.16, 2.75]
Kim (East Asian Study)	37	179	17	174	4.8%	2.12 [1.24, 3.61]
Liu	34	44	32	46	8.9%	1.11 [0.87, 1.43]
Marth (TRINOVA-2)	67	113	62	108	9.3%	1.03 [0.83, 1.29]
Pignata (MITO 11)	14	37	13	36	4.2%	1.05 [0.58, 1.91]
Pignata (MITO16B)	110	201	96	200	9.8%	1.14 [0.94, 1.38]
Richardson	34	50	30	50	8.1%	1.13 [0.84, 1.52]
Vergote (TRINOVA-3)	408	675	88	336	9.8%	2.31 [1.91, 2.79]
**Total (95% CI)**		**2789**		**1925**	**100.0%**	**1.28 [1.10, 1.49]**
Total events	1620		827			

Heterogeneity: Tau² = 0.05; Chi² = 62.82, df = 12 (P < 0.00001); I² = 81%

Test for overall effect: Z = 3.17 (P = 0.002)

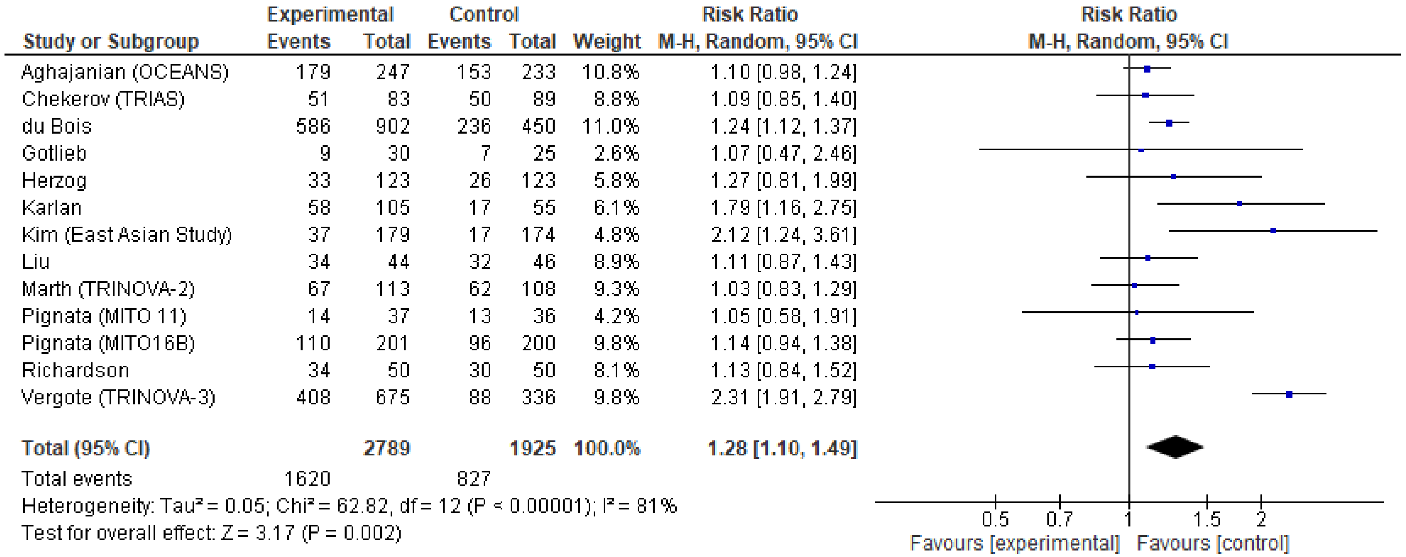

Figure S18: Vomiting

Study or Subgroup	Experimental Events	Experimental Total	Control Events	Control Total	Weight	Risk Ratio M-H, Random, 95% CI
Chekerov (TRIAS)	33	83	36	89	10.4%	0.98 [0.68, 1.42]
du Bois	406	902	126	450	16.8%	1.61 [1.36, 1.90]
Gotlieb	16	30	13	25	7.3%	1.03 [0.62, 1.70]
Herzog	21	123	17	123	5.9%	1.24 [0.69, 2.23]
Karlan	28	105	9	55	4.8%	1.63 [0.83, 3.21]
Kim (East Asian Study)	13	179	7	174	3.1%	1.81 [0.74, 4.42]
Liu	9	44	8	46	3.3%	1.18 [0.50, 2.77]
Marth (TRINOVA-2)	51	113	36	108	11.3%	1.35 [0.97, 1.89]
Pignata (MITO 11)	11	37	6	36	3.1%	1.78 [0.74, 4.31]
Pignata (MITO16B)	44	201	42	200	10.2%	1.04 [0.72, 1.52]
Pujade-Lauraine (AURELIA)	2	179	9	181	1.2%	0.22 [0.05, 1.03]
Richardson	21	50	13	50	6.2%	1.62 [0.91, 2.86]
Vergote (TRINOVA-3)	247	675	119	336	16.5%	1.03 [0.87, 1.23]
**Total (95% CI)**		**2721**		**1873**	**100.0%**	**1.24 [1.04, 1.47]**
Total events	902		441			

Heterogeneity: Tau² = 0.04; Chi² = 24.41, df = 12 (P = 0.02); I² = 51%
Test for overall effect: Z = 2.45 (P = 0.01)

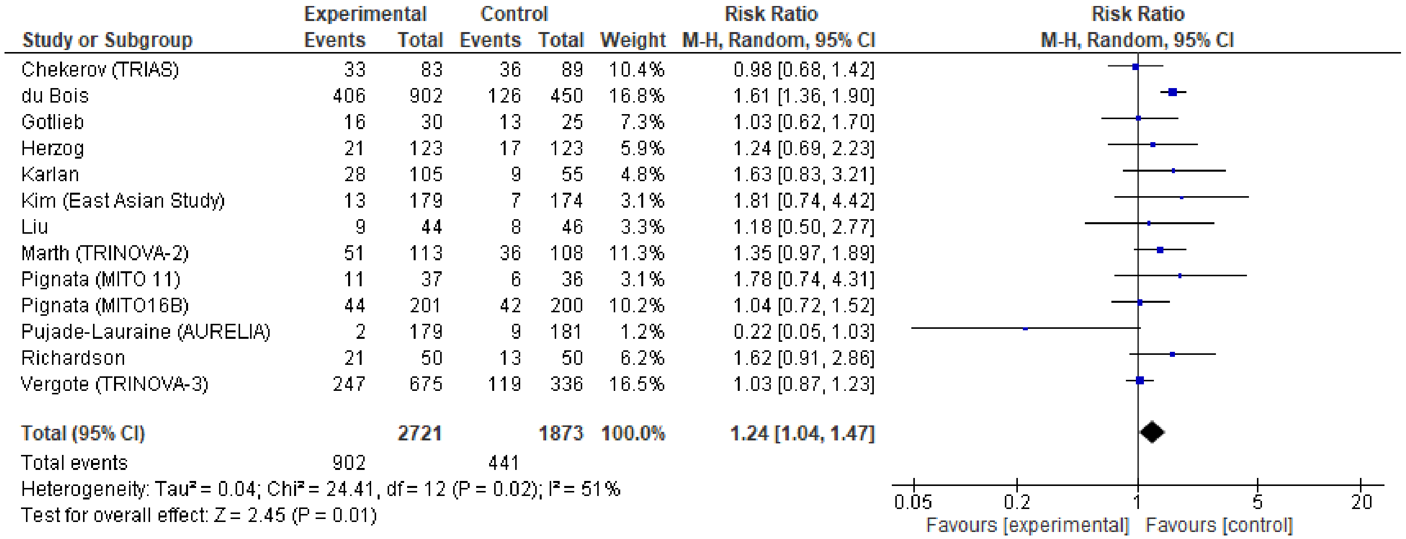

Figure S19: Anorexia

Study or Subgroup	Experimental Events	Experimental Total	Control Events	Control Total	Weight	Risk Ratio M-H, Random, 95% CI
Gotlieb	7	30	7	25	13.2%	0.83 [0.34, 2.06]
Herzog	20	123	5	123	12.8%	4.00 [1.55, 10.32]
Liu	13	44	4	46	12.0%	3.40 [1.20, 9.63]
Marth (TRINOVA-2)	27	113	23	108	16.7%	1.12 [0.69, 1.83]
Pignata (MITO 11)	9	37	5	36	12.4%	1.75 [0.65, 4.72]
Pignata (MITO16B)	21	201	9	200	14.5%	2.32 [1.09, 4.94]
Vergote (TRINOVA-3)	187	675	146	336	18.5%	0.64 [0.54, 0.76]
**Total (95% CI)**		**1223**		**874**	**100.0%**	**1.53 [0.84, 2.78]**
Total events	284		199			

Heterogeneity: Tau² = 0.49; Chi² = 38.98, df = 6 (P < 0.00001); I² = 85%
Test for overall effect: Z = 1.40 (P = 0.16)

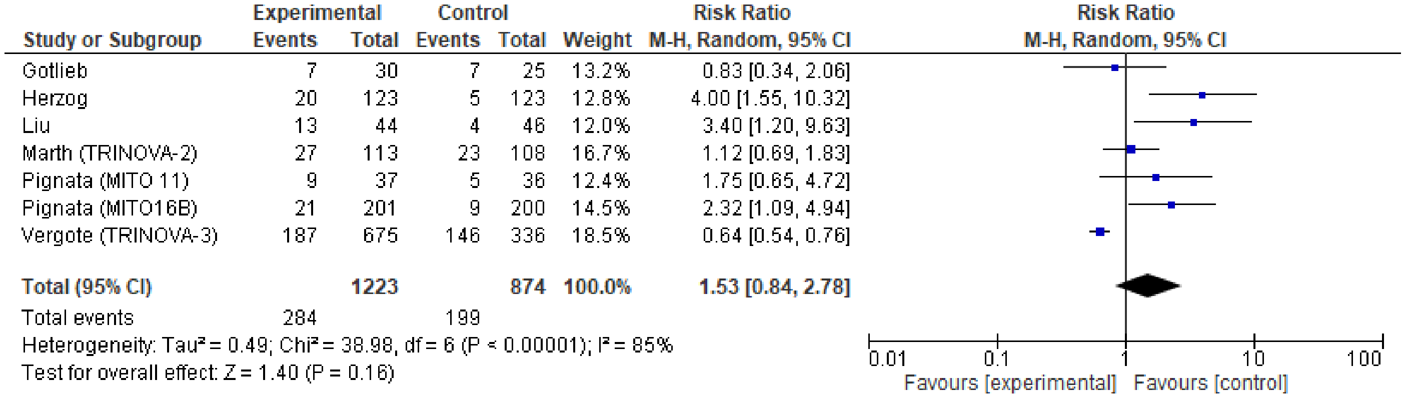

Figure S20: Diarrhea

Study or Subgroup	Experimental Events	Experimental Total	Control Events	Control Total	Weight	Risk Ratio M-H, Random, 95% CI
Aghajanian (OCEANS)	95	247	68	233	7.9%	1.32 [1.02, 1.70]
Chekerov (TRIAS)	33	83	26	89	7.0%	1.36 [0.90, 2.07]
du Bois	699	902	116	450	8.2%	3.01 [2.56, 3.53]
Gore	13	26	11	24	6.1%	1.09 [0.61, 1.95]
Gotlieb	13	30	2	25	2.6%	5.42 [1.35, 21.77]
Herzog	47	123	21	123	6.9%	2.24 [1.43, 3.51]
Karlan	41	105	15	55	6.6%	1.43 [0.87, 2.34]
Kim (East Asian Study)	73	179	20	174	6.9%	3.55 [2.27, 5.56]
Ledermann (ICON6)	86	95	65	115	8.2%	1.60 [1.35, 1.90]
Liu	41	44	1	46	1.6%	42.86 [6.16, 298.33]
Marth (TRINOVA-2)	33	113	28	108	7.0%	1.13 [0.73, 1.73]
Pignata (MITO 11)	21	37	7	36	5.3%	2.92 [1.42, 6.01]
Pignata (MITO16B)	39	201	19	200	6.5%	2.04 [1.22, 3.41]
Pujade-Lauraine (AURELIA)	5	179	5	181	3.1%	1.01 [0.30, 3.43]
Vergote (AGO-OVAR16)	253	477	80	461	8.0%	3.06 [2.46, 3.79]
Vergote (TRINOVA-3)	256	675	121	336	8.2%	1.05 [0.89, 1.25]
**Total (95% CI)**		**3516**		**2656**	**100.0%**	**1.92 [1.46, 2.53]**
Total events	1748		605			

Heterogeneity: Tau² = 0.23; Chi² = 149.64, df = 15 (P < 0.00001); I² = 90%

Test for overall effect: Z = 4.69 (P < 0.00001)

Risk Ratio
M-H, Random, 95% CI

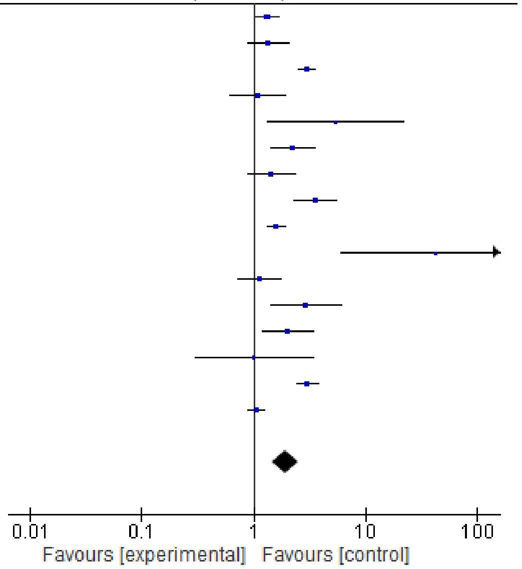

Figure S21: Constipation

Study or Subgroup	Experimental Events	Experimental Total	Control Events	Control Total	Weight	Risk Ratio M-H, Fixed, 95% CI
Chekerov (TRIAS)	31	83	30	89	8.1%	1.11 [0.74, 1.66]
Gore	1	24	0	26	0.1%	3.24 [0.14, 75.91]
Herzog	24	123	18	123	5.0%	1.33 [0.76, 2.33]
Karlan	34	105	9	55	3.3%	1.98 [1.02, 3.82]
Liu	8	44	2	46	0.5%	4.18 [0.94, 18.62]
Marth (TRINOVA-2)	39	113	35	108	10.0%	1.06 [0.73, 1.55]
Pignata (MITO 11)	11	37	12	36	3.4%	0.89 [0.45, 1.76]
Pignata (MITO16B)	64	201	57	200	16.0%	1.12 [0.83, 1.51]
Vergote (TRINOVA-3)	277	675	143	336	53.4%	0.96 [0.83, 1.12]
**Total (95% CI)**		**1405**		**1019**	**100.0%**	**1.08 [0.96, 1.21]**
Total events	489		306			

Heterogeneity: Chi² = 9.90, df = 8 (P = 0.27); I² = 19%
Test for overall effect: Z = 1.31 (P = 0.19)

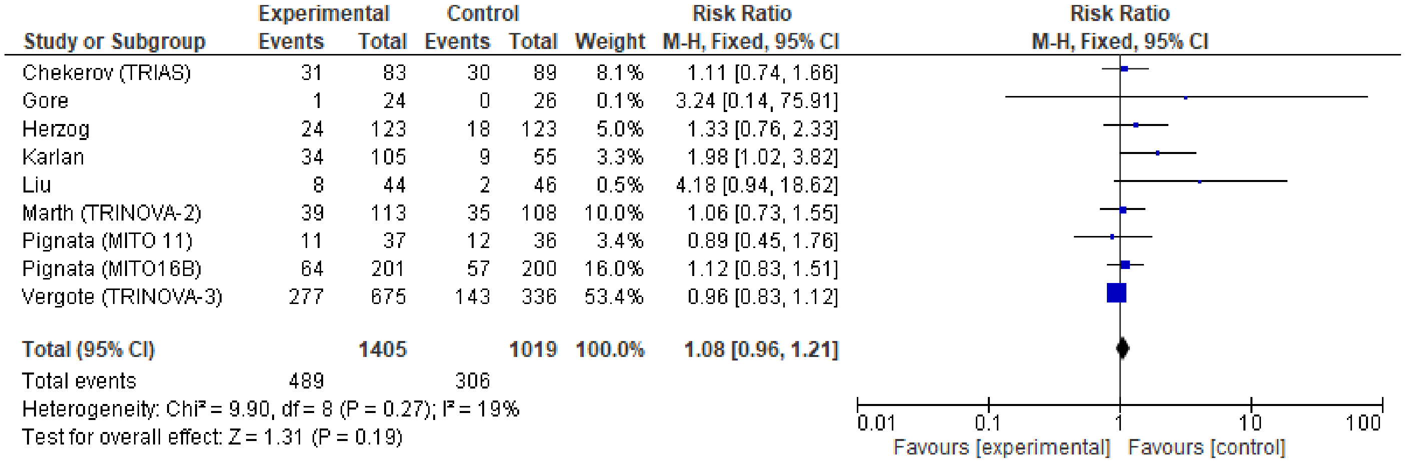

Figure S22: Fatigue

Study or Subgroup	Experimental Events	Experimental Total	Control Events	Control Total	Weight	Risk Ratio M-H, Fixed, 95% CI
Chekerov (TRIAS)	31	83	30	89	8.1%	1.11 [0.74, 1.66]
Gore	1	24	0	26	0.1%	3.24 [0.14, 75.91]
Herzog	24	123	18	123	5.0%	1.33 [0.76, 2.33]
Karlan	34	105	9	55	3.3%	1.98 [1.02, 3.82]
Liu	8	44	2	46	0.5%	4.18 [0.94, 18.62]
Marth (TRINOVA-2)	39	113	35	108	10.0%	1.06 [0.73, 1.55]
Pignata (MITO 11)	11	37	12	36	3.4%	0.89 [0.45, 1.76]
Pignata (MITO16B)	64	201	57	200	16.0%	1.12 [0.83, 1.51]
Vergote (TRINOVA-3)	277	675	143	336	53.4%	0.96 [0.83, 1.12]
**Total (95% CI)**		**1405**		**1019**	**100.0%**	**1.08 [0.96, 1.21]**
Total events	489		306			

Heterogeneity: Chi² = 9.90, df = 8 (P = 0.27); I² = 19%
Test for overall effect: Z = 1.31 (P = 0.19)

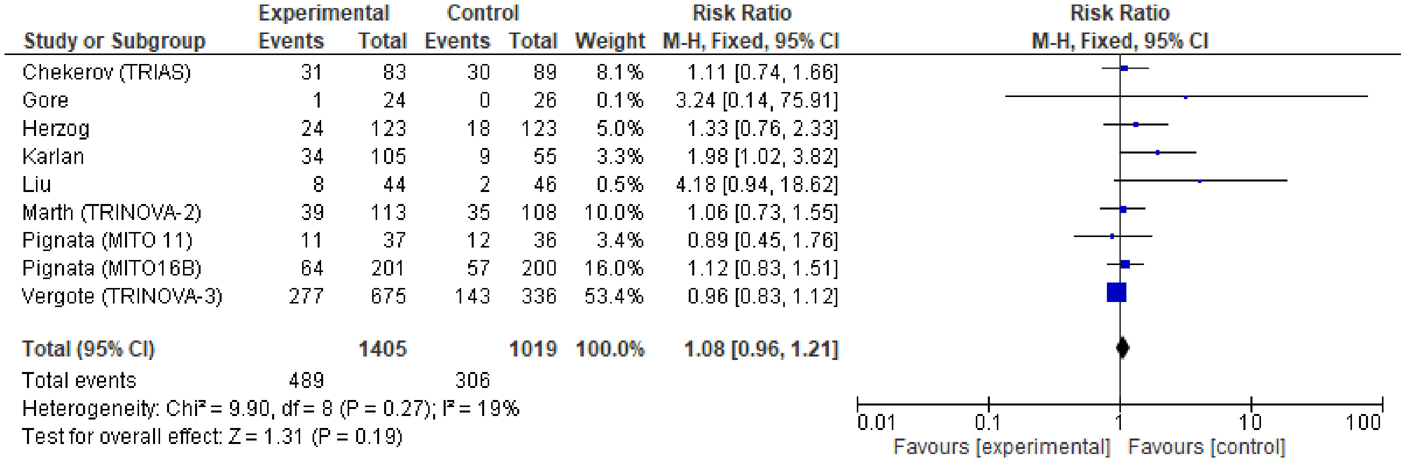

Figure S23: Dyspnea

Study or Subgroup	Experimental Events	Experimental Total	Control Events	Control Total	Weight	Risk Ratio M-H, Fixed, 95% CI
Aghajanian (OCEANS)	74	247	56	233	22.0%	1.25 [0.93, 1.68]
Chekerov (TRIAS)	22	83	17	89	6.3%	1.39 [0.79, 2.42]
du Bois	116	902	57	450	29.0%	1.02 [0.76, 1.37]
Gore	3	26	3	24	1.2%	0.92 [0.21, 4.14]
Gotlieb	13	30	7	25	2.9%	1.55 [0.73, 3.28]
Karlan	33	105	12	55	6.0%	1.44 [0.81, 2.56]
Liu	1	44	0	46	0.2%	3.13 [0.13, 74.93]
Marth (TRINOVA-2)	24	113	18	108	7.0%	1.27 [0.73, 2.21]
Pignata (MITO16B)	8	201	5	200	1.9%	1.59 [0.53, 4.78]
Pujade-Lauraine (AURELIA)	3	179	8	181	3.0%	0.38 [0.10, 1.41]
Vergote (TRINOVA-3)	125	675	40	336	20.4%	1.56 [1.12, 2.17]
**Total (95% CI)**		**2605**		**1747**	**100.0%**	**1.25 [1.08, 1.45]**
Total events	422		223			

Heterogeneity: Chi² = 8.10, df = 10 (P = 0.62); I² = 0%

Test for overall effect: Z = 2.98 (P = 0.003)

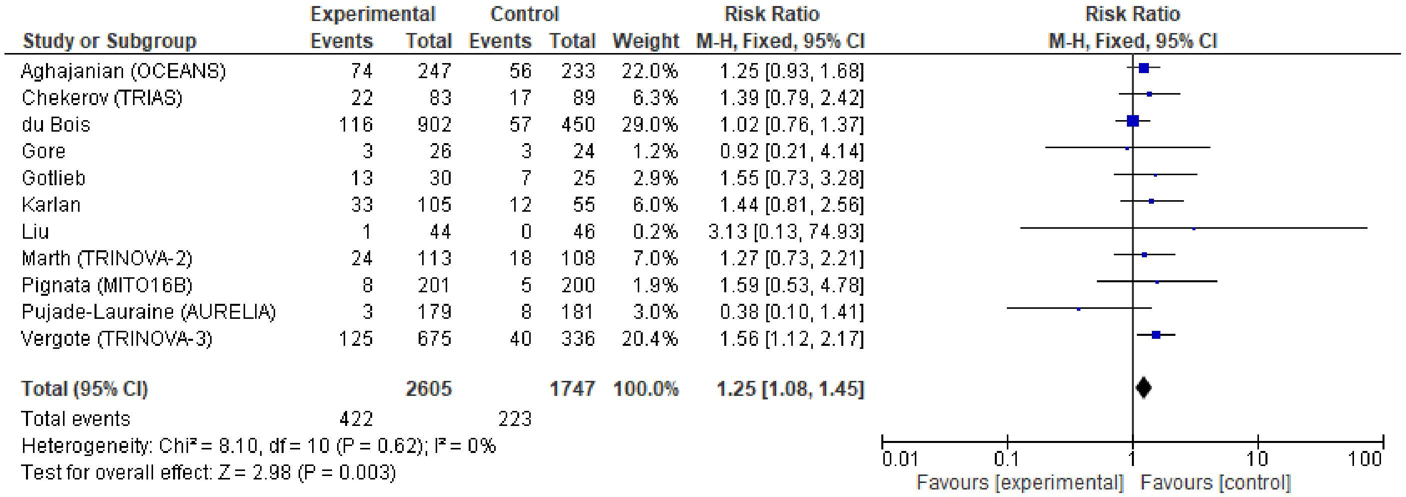

Figure S24: Alopecia

Study or Subgroup	Experimental Events	Experimental Total	Control Events	Control Total	Weight	Risk Ratio M-H, Fixed, 95% CI
Chekerov (TRIAS)	46	83	47	89	13.0%	1.05 [0.80, 1.38]
Gore	14	26	12	24	3.6%	1.08 [0.63, 1.84]
Herzog	13	123	6	123	1.7%	2.17 [0.85, 5.52]
Karlan	46	105	20	55	7.5%	1.20 [0.80, 1.82]
Marth (TRINOVA-2)	21	113	12	108	3.5%	1.67 [0.87, 3.23]
Pignata (MITO 11)	7	37	7	36	2.0%	0.97 [0.38, 2.50]
Vergote (TRINOVA-3)	363	675	179	336	68.6%	1.01 [0.89, 1.14]
**Total (95% CI)**		**1162**		**771**	**100.0%**	**1.07 [0.97, 1.19]**
Total events	510		283			

Heterogeneity: Chi² = 5.27, df = 6 (P = 0.51); I² = 0%

Test for overall effect: Z = 1.34 (P = 0.18)

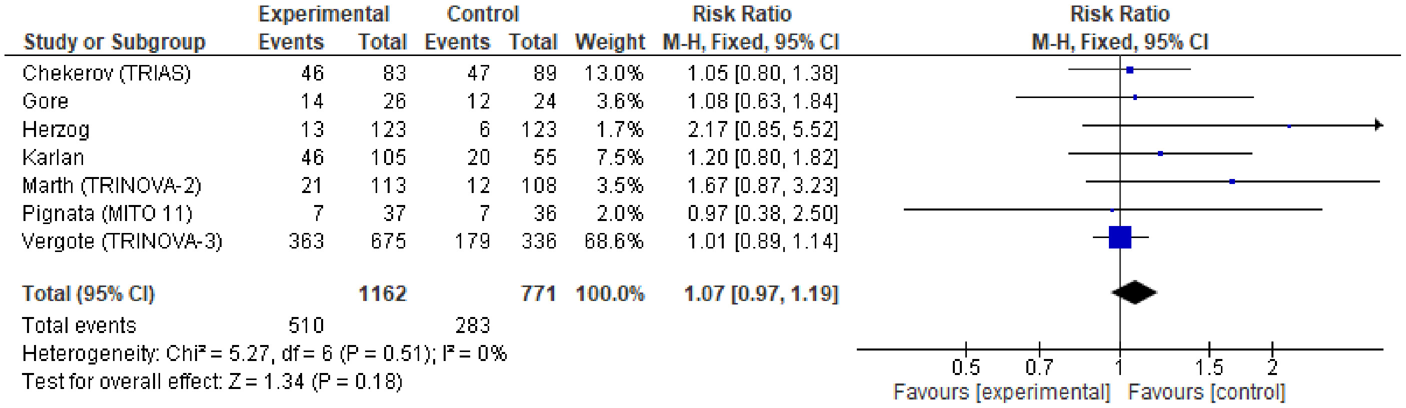

Figure S25: Rash

Study or Subgroup	Experimental Events	Experimental Total	Control Events	Control Total	Weight	Risk Ratio M-H, Random, 95% CI
Coleman (GOG0213)	295	330	278	327	27.5%	1.05 [0.99, 1.12]
Gore	7	26	6	24	14.7%	1.08 [0.42, 2.75]
Gotlieb	3	30	1	25	4.7%	2.50 [0.28, 22.56]
Herzog	62	123	17	123	22.5%	3.65 [2.27, 5.86]
Marth (TRINOVA-2)	2	113	2	108	5.8%	0.96 [0.14, 6.67]
Pignata (MITO 11)	0	37	0	36		Not estimable
Vergote (TRINOVA-3)	76	675	49	336	24.8%	0.77 [0.55, 1.08]
**Total (95% CI)**		**1334**		**979**	**100.0%**	**1.34 [0.79, 2.27]**
Total events	445		353			

Heterogeneity: $Tau^2 = 0.26$; $Chi^2 = 34.10$, df = 5 ($P < 0.00001$); $I^2 = 85\%$
Test for overall effect: Z = 1.09 (P = 0.28)

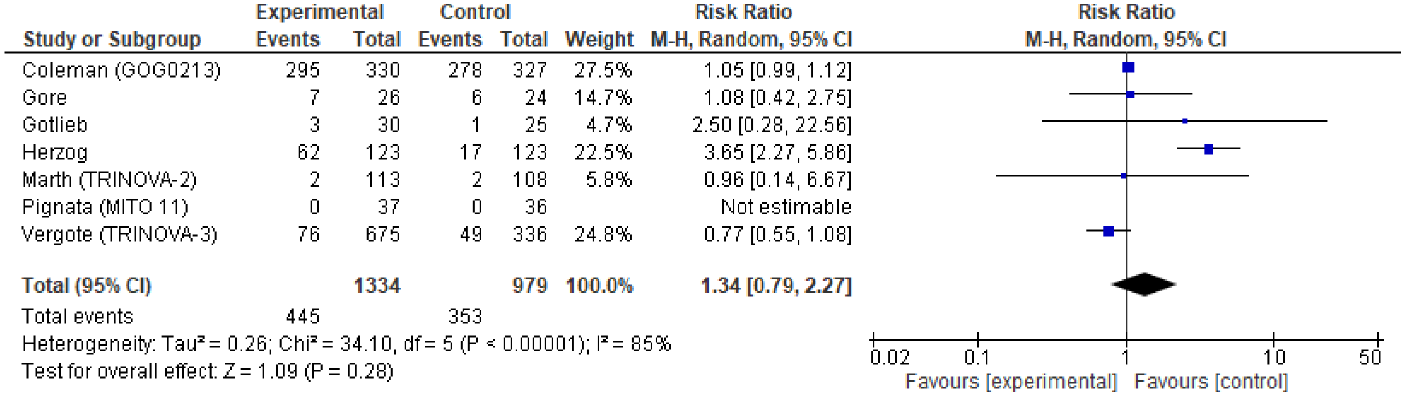

Figure S26: Hypomagnesemia

Study or Subgroup	Experimental Events	Experimental Total	Control Events	Control Total	Weight	Risk Ratio M-H, Random, 95% CI
Gore	3	26	1	24	12.2%	2.77 [0.31, 24.85]
Marth (TRINOVA-2)	11	113	3	108	27.2%	3.50 [1.00, 12.22]
Vergote (TRINOVA-3)	97	675	44	336	60.6%	1.10 [0.79, 1.53]
**Total (95% CI)**		**814**		**468**	**100.0%**	**1.69 [0.72, 3.93]**
Total events	111		48			

Heterogeneity: $Tau^2 = 0.28$; $Chi^2 = 3.70$, df = 2 (P = 0.16); $I^2 = 46\%$
Test for overall effect: Z = 1.21 (P = 0.23)

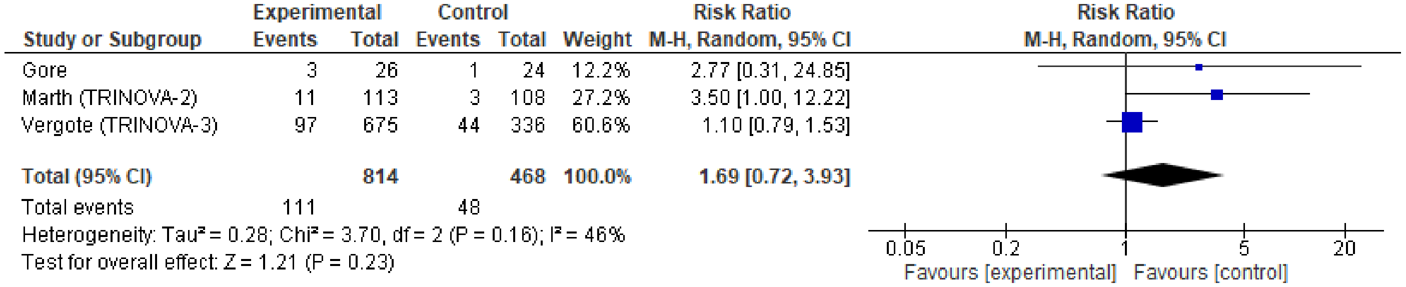

Figure S27: Hypokalemia

Study or Subgroup	Experimental Events	Experimental Total	Control Events	Control Total	Weight	Risk Ratio M-H, Fixed, 95% CI
Chekerov (TRIAS)	6	83	2	89	2.6%	3.22 [0.67, 15.49]
Karlan	19	105	3	55	5.4%	3.32 [1.03, 10.72]
Marth (TRINOVA-2)	24	113	11	108	15.4%	2.09 [1.07, 4.05]
Vergote (TRINOVA-3)	146	675	42	336	76.6%	1.73 [1.26, 2.38]
**Total (95% CI)**		**976**		**588**	**100.0%**	**1.91 [1.45, 2.51]**
Total events	195		58			

Heterogeneity: Chi² = 1.71, df = 3 (P = 0.63); I² = 0%
Test for overall effect: Z = 4.64 (P < 0.00001)

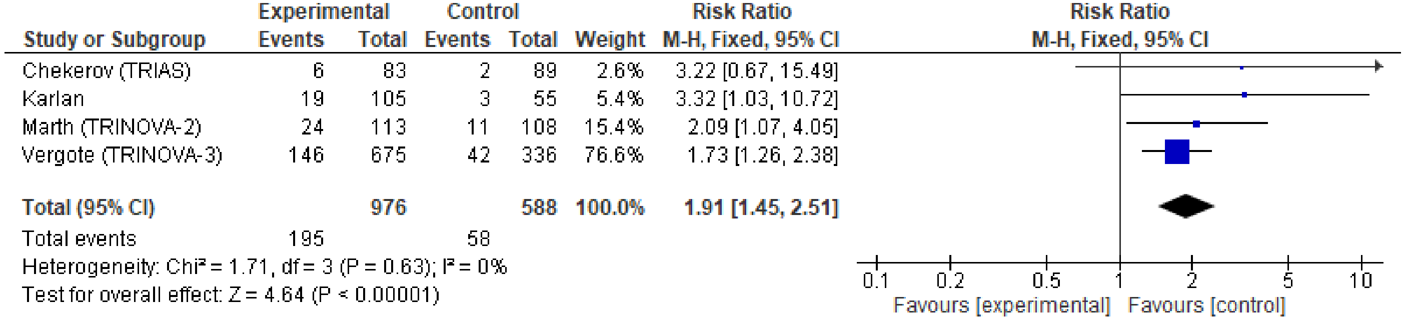

Figure S28: Pain

Study or Subgroup	Experimental Events	Experimental Total	Control Events	Control Total	Weight	Risk Ratio M-H, Fixed, 95% CI
Chekerov (TRIAS)	62	83	59	89	15.6%	1.13 [0.93, 1.37]
Coleman (GOG0213)	272	330	241	327	66.4%	1.12 [1.03, 1.21]
Gore	15	26	15	24	4.3%	0.92 [0.59, 1.45]
Pignata (MITO 11)	10	37	6	36	1.7%	1.62 [0.66, 4.00]
Vergote (TRINOVA-3)	72	675	33	336	12.1%	1.09 [0.73, 1.61]
**Total (95% CI)**		**1151**		**812**	**100.0%**	**1.12 [1.03, 1.21]**
Total events	431		354			

Heterogeneity: Chi² = 1.37, df = 4 (P = 0.85); I² = 0%
Test for overall effect: Z = 2.63 (P = 0.009)

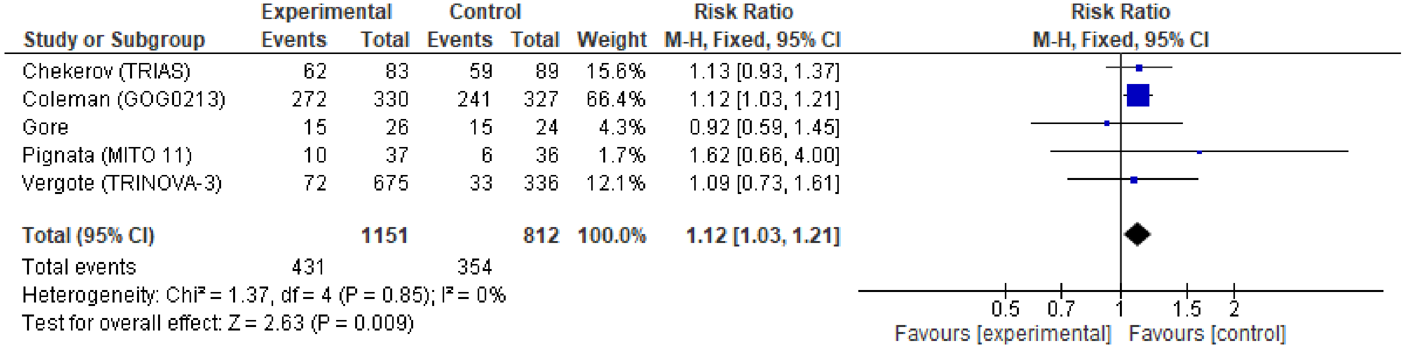

Figure S29: Headache

Study or Subgroup	Experimental Events	Experimental Total	Control Events	Control Total	Weight	Risk Ratio M-H, Random, 95% CI
Aghajanian (OCEANS)	120	247	70	233	16.3%	1.62 [1.28, 2.04]
Gore	9	26	7	24	6.3%	1.19 [0.52, 2.69]
Gotlieb	7	30	1	25	1.4%	5.83 [0.77, 44.28]
Herzog	28	123	19	123	10.4%	1.47 [0.87, 2.49]
Karlan	28	105	7	55	6.9%	2.10 [0.98, 4.49]
Kim (East Asian Study)	34	179	17	174	10.1%	1.94 [1.13, 3.35]
Liu	20	44	4	46	4.8%	5.23 [1.94, 14.08]
Marth (TRINOVA-2)	15	113	16	108	8.3%	0.90 [0.47, 1.72]
Pignata (MITO16B)	26	201	4	200	4.5%	6.47 [2.30, 18.19]
Vergote (AGO-OVAR16)	136	477	70	461	15.8%	1.88 [1.45, 2.43]
Vergote (TRINOVA-3)	125	675	55	336	15.2%	1.13 [0.85, 1.51]
**Total (95% CI)**		**2220**		**1785**	**100.0%**	**1.73 [1.34, 2.22]**
Total events	548		270			

Heterogeneity: Tau² = 0.09; Chi² = 25.93, df = 10 (P = 0.004); I² = 61%
Test for overall effect: Z = 4.25 (P < 0.0001)

Risk Ratio
M-H, Random, 95% CI

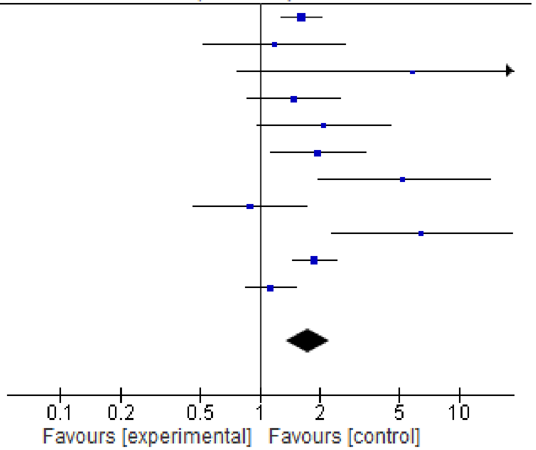

Figure S30: Abdominal pain

Study or Subgroup	Experimental Events	Experimental Total	Control Events	Control Total	Weight	Risk Ratio M-H, Fixed, 95% CI
Chekerov (TRIAS)	30	83	31	89	6.3%	1.04 [0.69, 1.55]
Gotlieb	7	30	5	25	1.1%	1.17 [0.42, 3.23]
Herzog	22	123	21	123	4.4%	1.05 [0.61, 1.80]
Karlan	34	105	18	55	5.0%	0.99 [0.62, 1.58]
Kim (East Asian Study)	22	179	20	174	4.3%	1.07 [0.61, 1.89]
Liu	9	44	3	46	0.6%	3.14 [0.91, 10.83]
Marth (TRINOVA-2)	35	113	41	108	8.8%	0.82 [0.57, 1.18]
Pignata (MITO16B)	23	201	16	200	3.4%	1.43 [0.78, 2.63]
Pujade-Lauraine (AURELIA)	5	179	10	181	2.1%	0.51 [0.18, 1.45]
Vergote (AGO-OVAR16)	169	477	142	461	30.3%	1.15 [0.96, 1.38]
Vergote (TRINOVA-3)	241	675	121	336	33.9%	0.99 [0.83, 1.18]
**Total (95% CI)**		**2209**		**1798**	**100.0%**	**1.05 [0.95, 1.17]**
Total events	597		428			

Heterogeneity: Chi² = 9.15, df = 10 (P = 0.52); I² = 0%
Test for overall effect: Z = 0.96 (P = 0.34)

Risk Ratio
M-H, Fixed, 95% CI

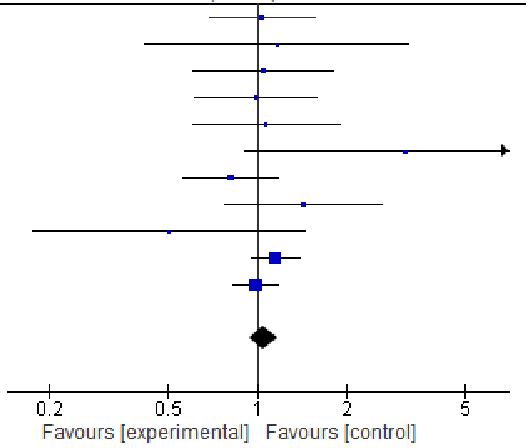

Figure S31: Back pain

Study or Subgroup	Experimental Events	Experimental Total	Control Events	Control Total	Weight	Risk Ratio M-H, Random, 95% CI	Risk Ratio M-H, Random, 95% CI
Aghajanian (OCEANS)	51	247	31	233	24.2%	1.55 [1.03, 2.34]	
Herzog	10	123	16	123	15.0%	0.63 [0.30, 1.32]	
Karlan	25	105	10	55	17.1%	1.31 [0.68, 2.53]	
Marth (TRINOVA-2)	11	113	18	108	16.1%	0.58 [0.29, 1.18]	
Vergote (TRINOVA-3)	92	675	60	336	27.6%	0.76 [0.57, 1.03]	
**Total (95% CI)**		**1263**		**855**	**100.0%**	**0.92 [0.62, 1.37]**	
Total events	189		135				

Heterogeneity: Tau² = 0.12; Chi² = 11.53, df = 4 (P = 0.02); I² = 65%
Test for overall effect: Z = 0.39 (P = 0.70)

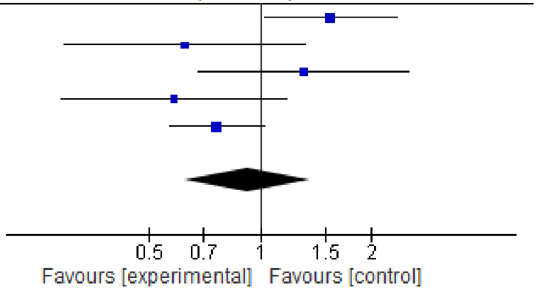

Supplementary Material 2 Prisma Checklist

Section/topic	#	Checklist item	Reported on page #
**TITLE**			
Title	1	Identify the report as a systematic review, meta-analysis, or both.	1
**ABSTRACT**			
Structured summary	2	Provide a structured summary including, as applicable: background; objectives; data sources; study eligibility criteria, participants, and interventions; study appraisal and synthesis methods; results; limitations; conclusions and implications of key findings; systematic review registration number or statement of absence of registration.	1,3
**INTRODUCTION**			
Rationale	3	Describe the rationale for the review in the context of what is already known.	2
Objectives	4	Provide an explicit statement of questions being addressed with reference to participants, interventions, comparisons, outcomes, and study design (PICOS).	3
**METHODS**			
Protocol and registration	5	Indicate if a review protocol exists, if and where it can be accessed (e.g., Web address), and, if available, provide registration information including registration number.	3
Eligibility criteria	6	Specify study characteristics (e.g., PICOS, length of follow-up) and report characteristics (e.g., years considered, language, publication status) used as criteria for eligibility, giving rationale.	3
Information sources	7	Describe all information sources (e.g., databases with dates of coverage, contact with study authors to identify additional studies) in the search and date last searched.	3.4
Search	8	Present full electronic search strategy for at least one database, including any limits used, such that it could be repeated.	3
Study selection	9	State the process for selecting studies (i.e., screening, eligibility, included in systematic review, and, if applicable, included in the meta-analysis).	4
Data collection process	10	Describe method of data extraction from reports (e.g., piloted forms, independently, in duplicate) and any processes for obtaining and confirming data from investigators.	4
Data items	11	List and define all variables for which data were sought (e.g., PICOS, funding sources) and any assumptions and simplifications made.	4

Risk of bias in individual studies	12	Describe methods used for assessing risk of bias of individual studies (including specification of whether this was done at the study or outcome level), and how this information is to be used in any data synthesis.	4
Summary measures	13	State the principal summary measures (e.g., risk ratio, difference in means).	4,5
Synthesis of results	14	Describe the methods of handling data and combining results of studies, if done, including measures of consistency (e.g., I^2) for each meta-analysis.	4,5

Section/topic	#	Checklist item	Reported on page #
Risk of bias across studies	15	Specify any assessment of risk of bias that may affect the cumulative evidence (e.g., publication bias, selective reporting within studies).	4, Figure 2
Additional analyses	16	Describe methods of additional analyses (e.g., sensitivity or subgroup analyses, meta-regression), if done, indicating which were pre-specified.	5
**RESULTS**			
Study selection	17	Give numbers of studies screened, assessed for eligibility, and included in the review, with reasons for exclusions at each stage, ideally with a flow diagram.	4
Study characteristics	18	For each study, present characteristics for which data were extracted (e.g., study size, PICOS, follow-up period) and provide the citations.	Table 2. and Table 3.
Risk of bias within studies	19	Present data on risk of bias of each study and, if available, any outcome level assessment (see item 12).	Figure 2
Results of individual studies	20	For all outcomes considered (benefits or harms), present, for each study: (a) simple summary data for each intervention group (b) effect estimates and confidence intervals, ideally with a forest plot.	4,5<br>Figures 3-7<br>Supplementary material 1
Synthesis of results	21	Present results of each meta-analysis done, including confidence intervals and measures of consistency.	4,5<br>Figures 3-7
Risk of bias across studies	22	Present results of any assessment of risk of bias across studies (see Item 15).	6<br>Figure 2
Additional analysis	23	Give results of additional analyses, if done (e.g., sensitivity or subgroup analyses, meta-regression [see Item 16]).	6<br>Supplementary

			material 3
**DISCUSSION**			
Summary of evidence	24	Summarize the main findings including the strength of evidence for each main outcome; consider their relevance to key groups (e.g., healthcare providers, users, and policy makers).	23-24
Limitations	25	Discuss limitations at study and outcome level (e.g., risk of bias), and at review-level (e.g., incomplete retrieval of identified research, reporting bias).	23
Conclusions	26	Provide a general interpretation of the results in the context of other evidence, and implications for future research.	7,20-22
**FUNDING**			
Funding	27	Describe sources of funding for the systematic review and other support (e.g., supply of data); role of funders for the systematic review.	24

From: Moher D, Liberati A, Tetzlaff J, Altman DG, The PRISMA Group (2009). Preferred Reporting Items for Systematic Reviews and Meta-Analyses: The PRISMA Statement. PLoS Med 6(7): e1000097. doi:10.1371/journal.pmed1000097

For more information, visit: www.prisma-statement.org.

Supplementary Material 3 Graphical representation of statistical analysis

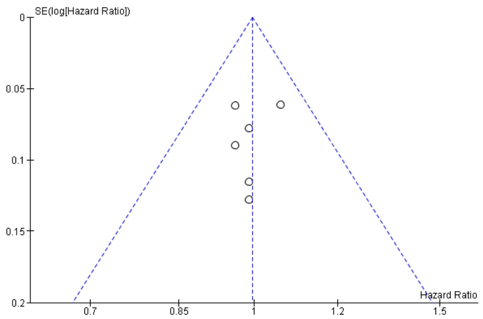

(a)

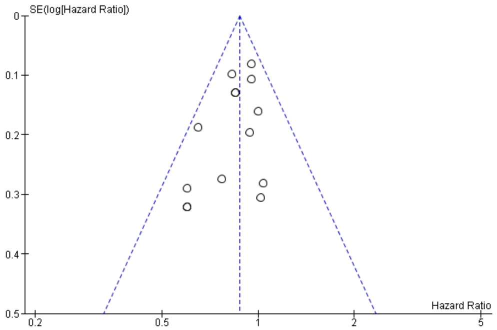

(b)

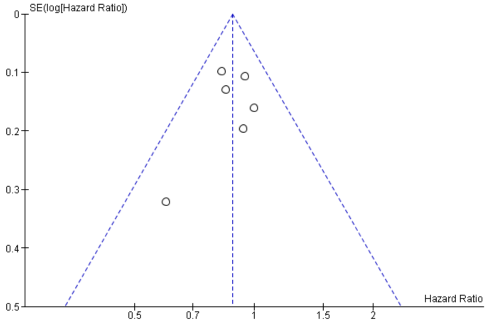

(c) (

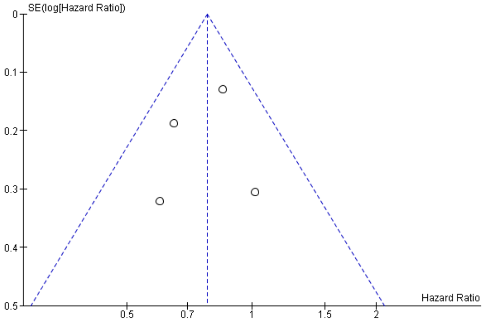

d)

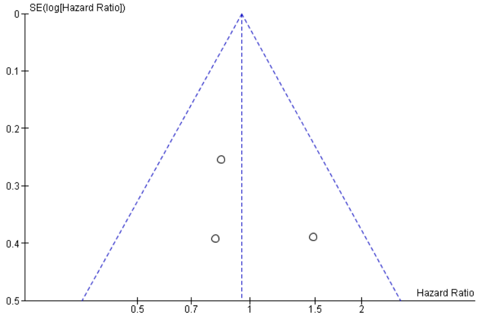

(e)

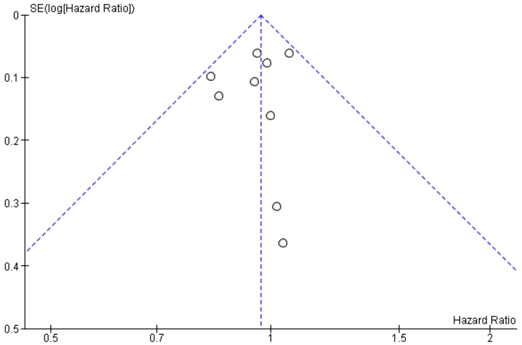

(f)

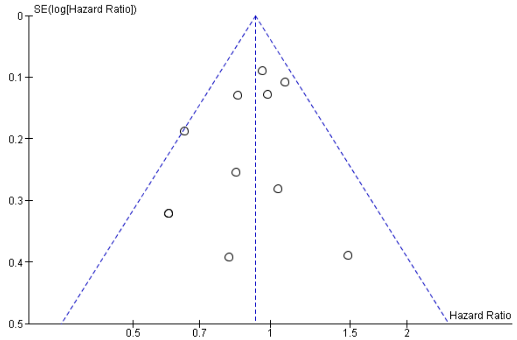

(g)

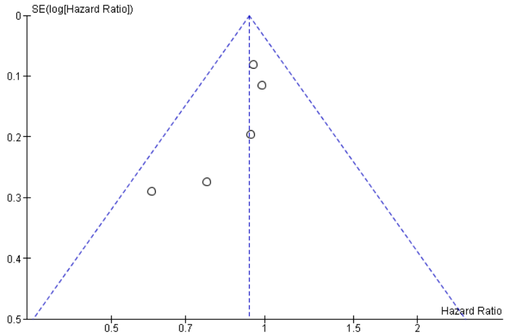

(h)

Figure S32 – Sensitivity analysis (OS): (a)first-line; (b)recurrent disease; (c)P-S R; (d) P-R R; (e)maintenance; (f)VEGF inhibitors; (g)VEGF-R inhibitors; (h)angiopoietin inhibitors

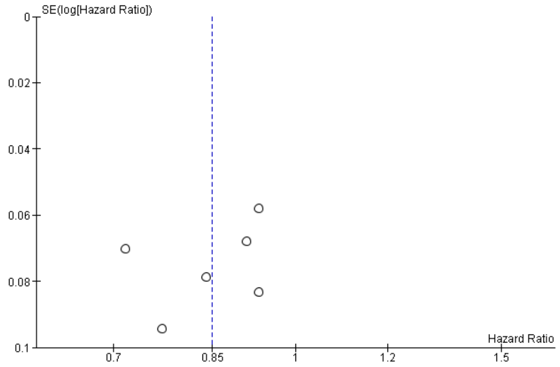
SE(log[Hazard Ratio])
0
0.02
0.04
0.06
0.08
0.1
Hazard Ratio
0.7
0.85
1
1.2
1.5
(a)

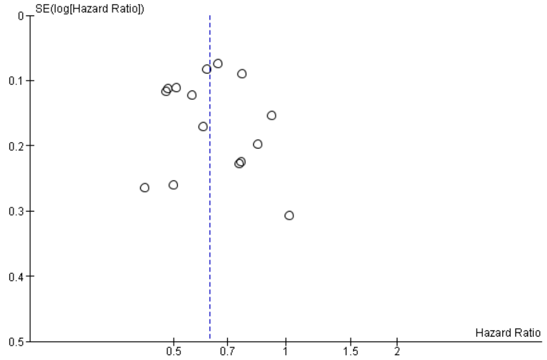
SE(log[Hazard Ratio])
0
0.1
0.2
0.3
0.4
0.5
Hazard Ratio
0.5
0.7
1
1.5
2
(b)

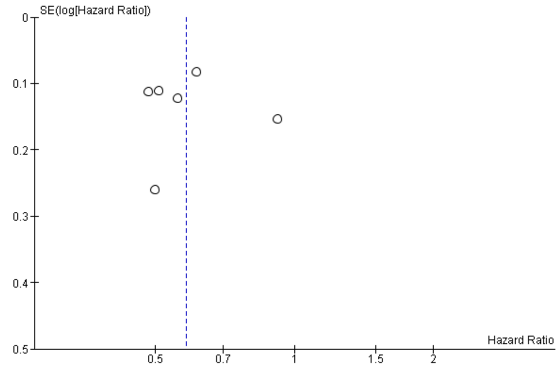
SE(log[Hazard Ratio])
0
0.1
0.2
0.3
0.4
0.5
Hazard Ratio
0.5
0.7
1
1.5
2
(c)

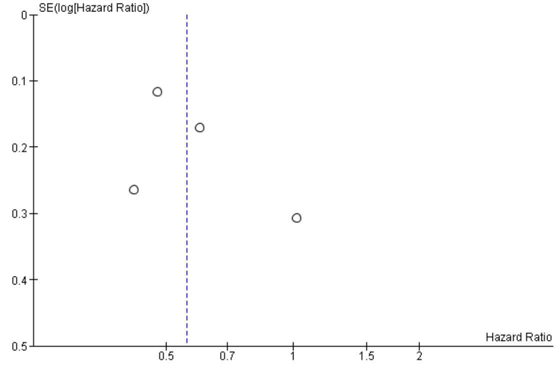
SE(log[Hazard Ratio])
0
0.1
0.2
0.3
0.4
0.5
Hazard Ratio
0.5
0.7
1
1.5
2
(d)

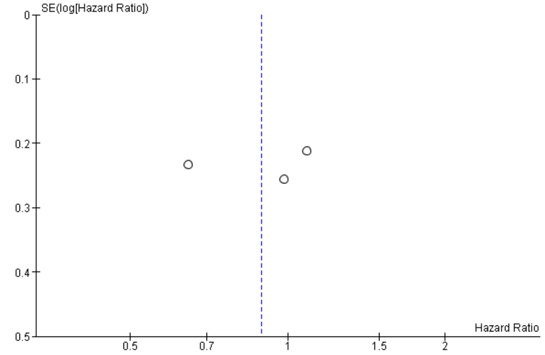
SE(log[Hazard Ratio])
0
0.1
0.2
0.3
0.4
0.5
Hazard Ratio
0.5
0.7
1
1.5
2
(e)

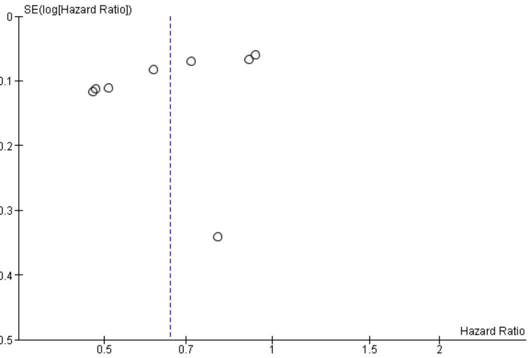
SE(log[Hazard Ratio])
0
0.1
0.2
0.3
0.4
0.5
Hazard Ratio
0.5
0.7
1
1.5
2
(f)

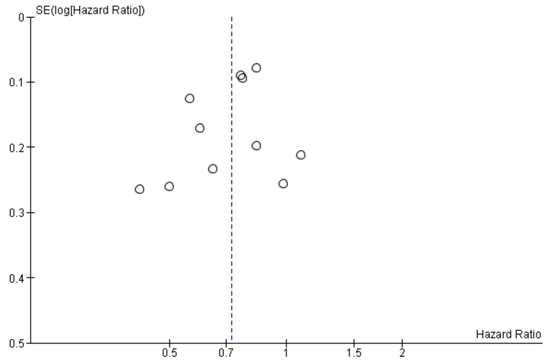

(g)

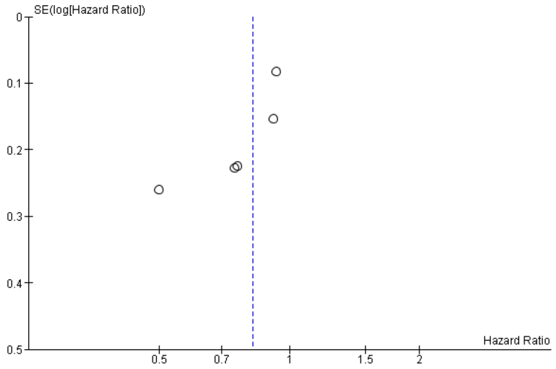

(h)

Figure S33 – Sensitivity analysis (PFS): (a)first-line; (b)recurrent disease; (c)P-S R; (d) P-R R; (e)maintenance; (f)VEGF inhibitors; (g)VEGF-R inhibitors; (h)angiopoietin inhibitors

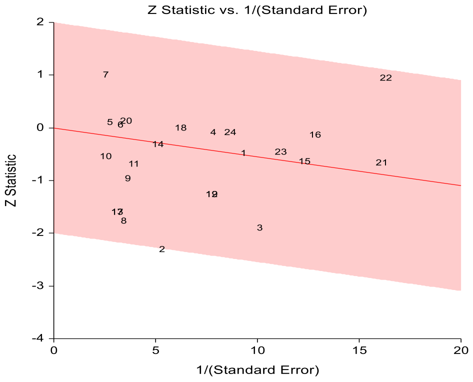
Z Statistic vs. 1/(Standard Error)
Z Statistic
1/(Standard Error)

(a)

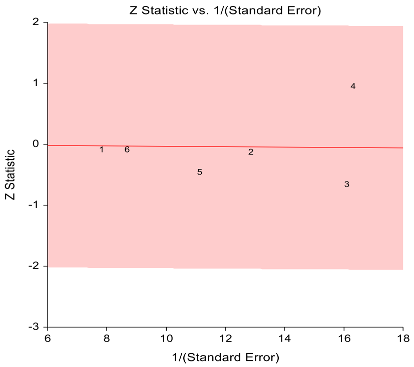
Z Statistic vs. 1/(Standard Error)
Z Statistic
1/(Standard Error)

(b)

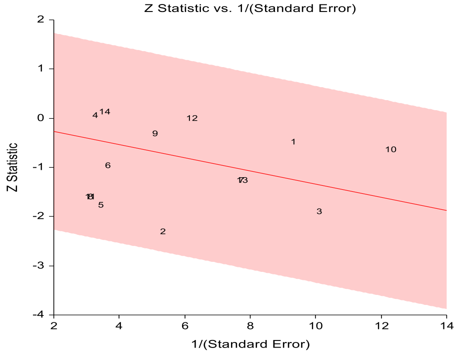
Z Statistic vs. 1/(Standard Error)
Z Statistic
1/(Standard Error)

(c)

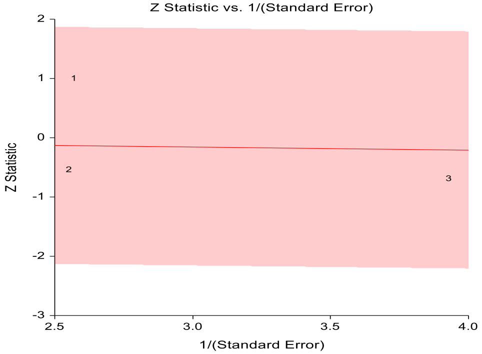
Z Statistic vs. 1/(Standard Error)
Z Statistic
1/(Standard Error)

(d)

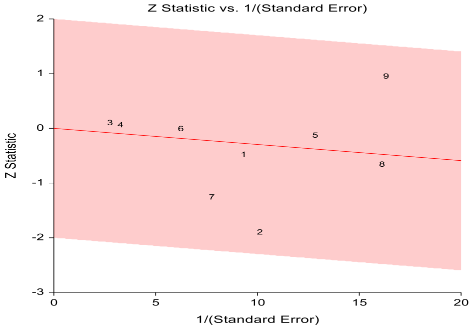
Z Statistic vs. 1/(Standard Error)
Z Statistic
1/(Standard Error)

(e)

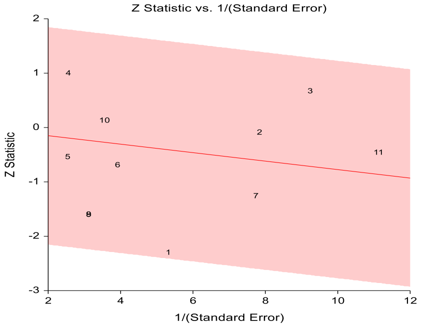
Z Statistic vs. 1/(Standard Error)
Z Statistic
1/(Standard Error)

(f)

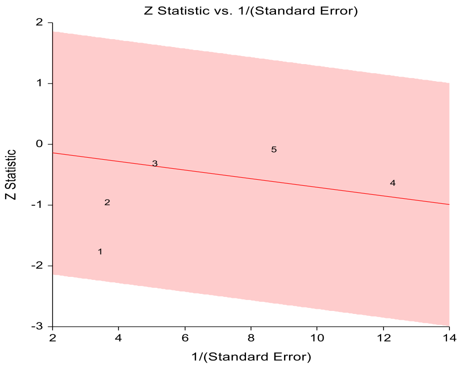

(g)

Figure S34 – Radial plots (OS): (a)pooled OS; (b)first-line; (c)recurrent disease; (d)maintenance; (e)VEGF inhibitors; (f)VEGF-R inhibitors; (g)angiopoietin inhibitors

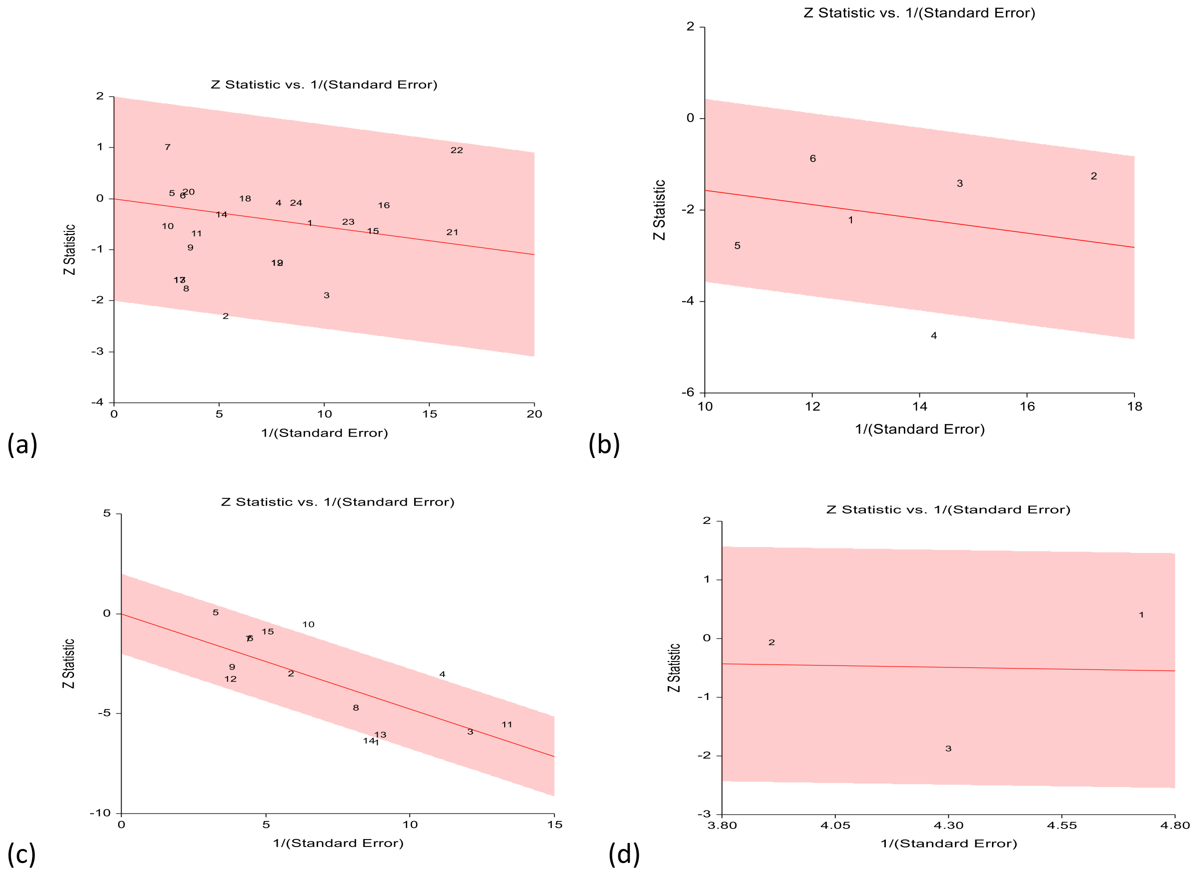
(a)
Z Statistic vs. 1/(Standard Error)
Z Statistic
1/(Standard Error)
(b)
Z Statistic vs. 1/(Standard Error)
Z Statistic
1/(Standard Error)
(c)
Z Statistic vs. 1/(Standard Error)
Z Statistic
1/(Standard Error)
(d)
Z Statistic vs. 1/(Standard Error)
Z Statistic
1/(Standard Error)

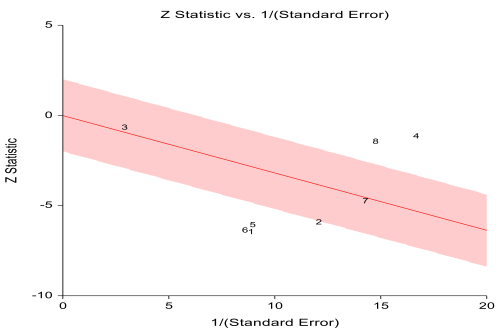

(e)

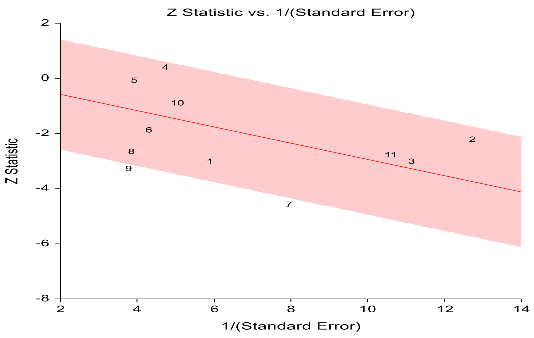

(f)

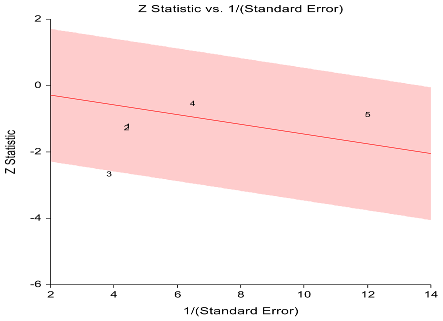

(g)

Figure S35 – Radial plots (PFS): (a)pooled PFS; (b)first-line; (c)recurrent disease; (d)maintenance; (e)VEGF inhibitors; (f)VEGF-R inhibitors; (g)angiopoietin inhibitors
